# Impact assessment of a national research collaboration improving health outcomes for working-age Australians with disability

**DOI:** 10.1186/s12961-026-01448-7

**Published:** 2026-02-17

**Authors:** Jodie Bailie, Helen Dickinson, Alex Sully, Dennis Petrie, Anne Kavanagh, Sophie Yates, Gwynnyth Llewellyn, Stefanie Dimov, Hannah Badland

**Affiliations:** 1 Centre for Disability Research and Policy, University of Sydney, Camperdown, Australia; 2 University Centre for Rural Health, University of Sydney, 61 Uralba Street, Lismore, NSW 2480 Australia; 3 School of Business, University of New South Wales, Canberra, Australia; 4 Melbourne School of Population and Global Health, University of Melbourne, Melbourne, Australia; 5 Centre for Health Economics, Monash University, Caulfield, VIC Australia; 6 Crawford School of Public Policy, Australian National University, Canberra, Australia; 7 Social Equity Research Centre, RMIT University, Melbourne, Australia

**Keywords:** Impact assessment, Disability, Research collaboration, Evaluation

## Abstract

**Background:**

Despite growing recognition of the need for cross-disciplinary research collaborations to tackle complex issues, the impact of such collaborations is rarely documented. This study applied an impact framework to assess the impact of a cross-disciplinary disability research collaboration, the Centre of Research Excellence in Disability and Health (CRE-DH) (2016–2023). We report on the utility of the impact framework for this task and propose a set of actions to ensure the effectiveness of impact evaluations for other cross-disciplinary collaborations.

**Methods:**

We retrospectively applied the Framework to Assess the Impact from Translational health research (FAIT) to the CRE-DH, which included a modified payback framework, an economic analysis and a narrative account of the impact generated by the collaboration. The impact assessment covered the period 2016–2024. Data were gathered from project records, reports and publications and secondary analysis of interviews conducted with external and internal stakeholders. Data were mapped to five impact domains: knowledge advancement, capacity strengthening, policy contribution, economic impact and community benefit.

**Results:**

The $5.4 million (2024 AUD) initially invested in the CRE-DH delivered an additional investment of $39.9 million (2024 AUD) (sensitivity analysis $26.5–53.2 million) in leveraged grants, consultancies and fellowships. Collectively, CRE-DH members produced 148 publications, 4 books, 15 book chapters, 48 reports, 17 fact sheets and 132 media articles. Publications were mentioned in the media more than 3400 times and cited in 45 policy documents. The CRE-DH funded and developed 9 early career disability researchers, and held 11 external events to strengthen capacity in disability research with policymakers and disability organizations. A total of 29 policy submissions were made, with those focussing on coronavirus disease 2019 (COVID-19) and its disproportionate impact on people with disability having particular influence on government policy.

**Conclusions:**

Applying FAIT retrospectively to assess a cross-disciplinary research collaboration’s impact allowed us to examine multiple benefit domains. The CRE-DH demonstrated impact in advancing knowledge, strengthening capacity, influencing policy and generating economic benefits; evidence on quantifiable community benefit is not yet available. However, our assessment was limited by insufficient data collection on some critical issues, such as disability representation. As a result, we propose several recommendations for applying FAIT in future research collaborations.

**Supplementary Information:**

The online version contains supplementary material available at 10.1186/s12961-026-01448-7.

## Introduction

Around 1.3 billion people (or 1 in 6) live with disability worldwide [1]. On average, people with disability experience poorer health than those without disability across a range of physical and mental health outcomes [1–3]. However, research shows that much of the health inequity between these groups is avoidable [4–6]. This is because the poorer health outcomes of people with disability often stem from social and economic disadvantage, as well as a lack of access and inclusion related to health services, health promotion and preventative care.

Cross-disciplinary research collaborations have been recommended as a way to gather and understand the research evidence needed to address the drivers of health inequity for people with disability [7]. However, for the value of this type of collaboration to be maximized, the outcomes of the research need to be translated into policy and practice to generate wider societal benefits [7]. This push for applied research has led to evaluation efforts that both measure the impact of research collaborations and identify opportunities for future policy influence. Despite a plethora of research impact frameworks and tools, including systematic reviews of these frameworks [8–10], we have been unable to identify any published impact assessments of cross-disciplinary research collaborations focussed on improving health outcomes for people with disability.

To address this gap, our study aims to assess the impact of the Centre of Research Excellence in Disability Health (CRE-DH) [11], a cross-disciplinary research collaboration that operated from 2016 to 2023. In doing so, we aim to inform ongoing efforts to improve health outcomes for people with disability through collaborative research endeavours and contribute to the broader literature on research collaboration impact. We also report on the utility of using a documented impact framework to evaluate a cross-disciplinary research collaboration.

### Study setting

**Fig. 1:**
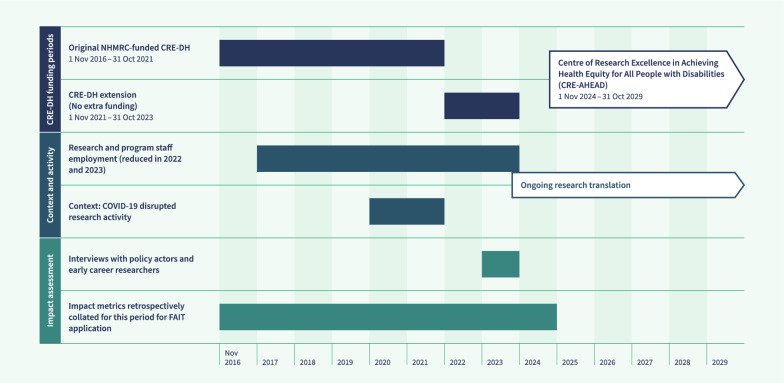
Overview of CRE-DH key dates

Australia’s National Health and Medical Research Council (NHMRC) funded the Centre of Research Excellence program in 2014 to improve Australians’ health outcomes through supporting translation of research into policy and practice, collaborative research and research capacity-strengthening activities. The CRE-DH was funded under this program for 5 years (1 November 2016–31 October 2021); however, due to coronavirus disease 2019 (COVID-19) delays and impacts it was extended without additional funding for another 2 years (to 31 October 2023). During this extension phase the focus shifted to advancing research translation, although there was some limited employment of research and program staff. This context and setting information is outlined in Fig. 1 and further discussed in relevant sections of this paper.

#### Research aims

**Fig. 2:**
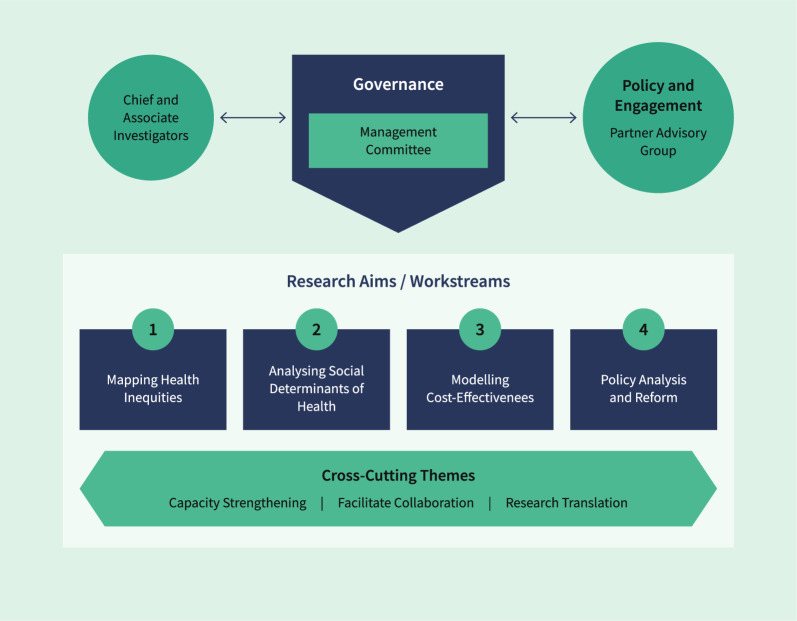
Governance and management structure of the CRE-DH

The goal of the CRE-DH was to identify implementable, cost-effective policy interventions to improve the health of Australians with disability aged 15–64 years. This was to be achieved both by strengthening research capacity and by bringing together a cross-disciplinary group of senior, mid-career and early career researchers and graduate students with skills in epidemiology, health economics, health and social policy, psychology, human geography and public health. The group addressed four workstreams across the following interconnected research objectives: (1) mapping the health inequities between Australians with and without disability; (2) analysing the social, economic and environmental factors that contribute to the poorer health of people with disability; (3) modelling the cost-effectiveness of health policy interventions; and (4) policy analysis and reform (Fig. 2).

#### Governance and management

The CRE-DH brought together investigators from the following Australian tertiary institutions: University of Melbourne, University of Sydney, University of New South Wales (UNSW), RMIT University, Monash University and Australian National University. International investigators came from the WHO, the University of York, Oregon State University and the London School of Hygiene & Tropical Medicine (see Appendix 1 for a full listing).

Governance of the CRE-DH (Fig. 2) was carried out by a Management Committee of five chief investigators. Operating as an open collaboration, the CRE-DH then actively invited other Australian researchers, organizations and agencies involved in improving health outcomes for people with disability to become affiliates of the Centre. As a result, the CRE-DH established an affiliate program offering early, mid-career and senior disability health researchers support, networking and collaboration opportunities, access to capacity-strengthening forums and seminars, and mentorship by senior researchers.

The Centre also set up a Partner Advisory Group to guide research priorities and outputs, as well as advise on engaging with stakeholders and ensuring the inclusion of people with disability in the work of the CRE-DH. This group consisted of senior members from government and nongovernment organizations, statutory bodies, peak bodies, consumer representative and advocacy organizations. The CRE-DH coordinating centre located at the University of Melbourne supported the CRE-DH’s four research workstreams.

## Method

### Study design

Following an evaluation of the CRE-DH [12], we undertook a retrospective mixed methods impact assessment that drew on the Framework to Assess the Impact from Translational health research (FAIT) [13–18]. A detailed description of the development of FAIT can be found elsewhere [10, 13]. In brief, FAIT is a hybrid of three validated methods for assessing research impact: (1) metrics based on modified payback; (2) economic analysis; and (3) narrative accounts of research benefits from the end-user perspective. These methods are underpinned by a program logic model.

FAIT was selected for the following reasons. First, its multidimensional lens was the best approach for conducting an impact assessment of the CRE-DH. Second, FAIT emphasizes translational health research, which aligns with the CRE-DH’s aspiration of influencing policy and practice. Third, although FAIT methodology has been applied to a range of Australian Centres of Research Excellence [17, 19, 20] and other research collaborations [14, 21], it had not yet been applied to a research collaboration in the disability context.

This research was approved by the UNSW Canberra Human Research Ethics Advisory Panel (#HC220696). A description of each phase as it was applied to the CRE-DH evaluation is given below. Although the CRE-DH finished in 2023, the impact assessment period covered 2016–2024 to capture any impacts that continued into the following year.

### Program logic model

We created a program logic model outlining the need that the CRE-DH aimed to address, the activities designed to meet that need, the outputs from those activities (including research translation) and the intended end users of these outputs (Table 1). Potential impacts were grouped into specific domain benefits [22].

**Table 1:** Retrospective program logic model for the application of FAIT to the impact assessment of the CRE-DH

Need (the need driving the CRE-DH and its activities)	• 1 in 6 Australians have a disability • People with disability generally experience poorer health than others across most health outcomes • The poor health of people with disability may be unrelated to their impairment • Inadequate evidence/knowledge about: ◦ the social and economic determinants of disability-related health inequities ◦ the health impacts on people with disability and the cost-effectiveness of social and health policies • Shortage of public health research capacity in the disability sector • Absence of a cross-sectoral approach
Research objectives (the overarching research objectives of the CRE-DH)	• Map the spatial distribution of social, economic and health inequities between people with and without disability • Apply cutting-edge epidemiological methods to establish the main social determinants of health for people with disability • Build the first ever cost-effectiveness model to estimate the health impacts and value for money of policy interventions for people with disability • Involve stakeholders in the co-production of knowledge
Activities (strategic initiatives to address aims, i.e., the things we did)	Research production • Provide seed funding to develop new research collaborations • Employ early career researchers (ECRs) as postdoctoral (postdoc) fellows • Embed stakeholders in the research process to co-produce knowledge with the explicit intent of policy reform • Use data integration platforms to identify and map the spatial distribution of social, economic and health inequities between Australians with and without disability • Build a cost-effectiveness model to estimate the health impacts and value of policy interventions for people with disability Capacity strengthening • Provide scholarship funding for PhD students • Conduct and host capacity-building activities for CRE-DH affiliates (PhDs, postdocs, ECRs) and others across research and policy sectors • Conduct workshops, webinars and forums to inform advocacy and policy development Research translation • Increase awareness of CRE-DH research with a dedicated website, social media presence and regular press releases • Develop fact sheets, reports, policy responses and submissions, webinars and workshops to inform policy development Governance and coordination • Establish a central coordinating point for the CRE-DH to operate virtually and to hold records and outputs • Hold regular leadership meetings Stakeholder engagement • Establish and implement a Partner Advisory Group • Undertake policy/parliamentary submissions to inform relevant inquiries and commissions
Outputs	• Publications, reports and fact sheets • Presentations, webinars, policy forums and workshops • Submissions and policy responses • Grant proposals* • Increased disability research capacity through postdocs and PhD students trained in disability research • Affiliated PhDs and master’s students • CRE-DH social media and website • Partner Advisory Group • Disability data development
Stakeholders/end users	• CRE-DH researchers, both national and international • Other disability and health researchers • Disability policy and advocacy organizations • People with disability and carers • Industry • Government, especially Commonwealth Departments of Health and of Social Services
Short-term impacts	Knowledge advancement • New knowledge on disability and health generated and disseminated, including academic publications, presentations, reports, social media, citations, downloads and collaboration Capacity and capability strengthened • Growing numbers of affiliated PhD completions and postdocs commencing • Increasing number and diversity of capacity-strengthening events • Meaningful participation of people with disability in research production and dissemination Economic impact • Grant funding, fellowships, awards and scholarships leveraged
Medium-term impacts	Informed policy • Strong relationships developed between researchers and research users • Input into developing disability-related policy through submissions, inquiries and invitations to present research findings • Citations in policy documents • Changes to policy because of submissions and advocacy
Long-term impacts	Economic benefit • Value of improved health outcomes for people with disability • Value of increased lifetime earnings post-PhD Community benefit • Reduced inequalities in health outcomes between people with and without disability

^*^Whilst FAIT considers future research grants as an economic outcome, we instead consider that the research program has been able to attract further investment as an intermediate outcome that may lead to improvements in the wellbeing of people with disability in the future

The logic model was designed through a review of CRE-DH documents (e.g. grant submission, reports, website, draft internal program logic), program logics from other retrospective FAIT applications and input from CRE-DH chief investigators and the authors of this paper.

### Payback

We used the payback framework [22] to identify CRE-DH impact by selecting appropriate quantifiable impact metrics in each of the relevant domains. This approach was a modification from the original payback framework used for qualitative assessments. Our process was iterative and included reviewing the previous evaluative work of the CRE-DH [12], other retrospective applications of FAIT [15, 16, 18] and metric databanks, such as Becker Medical Library Model for Assessment of Research Impact [23]. The impact metrics were grouped under the five payback domains relating to anticipated CRE-DH benefits: knowledge advancement; capacity strengthening; policy contribution; economic impact; and community benefit.

We set 31 December 2024 as the cutoff date for impact metrics related to CRE-DH outputs, such as publications, books, reports, translational articles and policy and parliamentary submissions (Fig. 1). This additional year after the CRE-DH concluded in 2023 allowed time for publications to be available in the public domain, and for additional translational activities and funding impacts to be realized. A cutoff date of 17 December 2024 was used for all publication statistics, views and citations of outputs, and for grants and fellowships, the cutoff was 31 December 2023.

Metric data were primarily sourced from CRE-DH project administrative records maintained by the coordinating centre in Melbourne, chief investigator diaries and curriculum vitae. Citations and views for publications and books were sourced from SciVal and Google Scholar. Altmetric was used to identify publication citations in policy documents and news outlets. Where required, costing data were obtained from published values.

CRE-DH administrative records underwent routine quality assurance processes prior to analysis. All data collected were screened for completeness and internal consistency, with standard cleaning procedures applied, including duplicate checks and author team review. Analyses were restricted to records with complete and verified data.

### Economic analysis

To understand the return on investment from the CRE-DH, we selected cost-consequence analysis as the most relevant and pragmatic method based on available data and the diverse nature of the outputs [24]. This presents a range of costs and consequences in a disaggregated form, allowing impacts to be reported transparently when monetization or aggregation into a single measure over a common time horizon (e.g. return on investment) is not feasible [24]. All monetary values were converted into their AUD value as of June 2024 using the Australian consumer price index [25].

#### Costs

The bulk of our research costs were covered by the main study grant from the NHMRC. In addition, we included in-kind contributions from chief investigators. Wage rates were sourced from the University of Sydney Enterprise Agreement (2024) [26], with 39% direct oncosts applied but indirect oncosts excluded. To estimate the value of in-kind contributions, we matched each chief investigator to their predominant academic level at the time of their CRE-DH involvement. In-kind time was estimated as follows: two chief investigators at 20% full-time equivalent (FTE), two at 10% FTE, and six at 5% FTE. In-kind time contributions from associate investigators and key stakeholders (e.g. representatives of policy or disability organizations) were excluded.

Most forums and workshops were held online so incurred minimal direct costs. We include costs for the six in-person events, but exclude travel time and costs paid from outside of the CRE-DH grant. In-kind time contributions for seminars, workshops and forums were costed when in-person attendee numbers were available, and were based on the average wage rate for a senior research fellow (step 3) at the University of Sydney (2024) [26], with 39% direct oncosts. On the basis of estimates from the chief investigators, as an average, we assumed 2 h of participation per meeting per day, recognizing that some meetings may have exceeded this duration (Appendix 3).

#### Consequences

For this study, we limited our assessment of consequences to funding leveraged because of the CRE-DH (an intermediate outcome, reported in AUD), and other outcomes such as publications that could be measured in their natural units (Table 2). On the basis of estimates from the chief investigators, we attributed 75% of that leveraged funding to the CRE-DH itself, that is, we assumed that only 25% of outcomes would have occurred in the absence of the CRE-DH. This attribution level reflects expert judgement by the chief investigators, who were best placed to assess the CRE-DH’s contribution to enabling these outcomes. The high attribution level likely reflects that CRE-DH brought together many researchers who had previously never worked with each other. We also undertook a sensitivity analysis that applied alternative attribution levels of 50% and 100% to capture potential minimum and maximum values.

**Table 2:** Impact metrics for the CRE-DH by the payback domains of benefit

Domains		Metric	Results
Knowledge advancement	Peer-reviewed publications, books and book chapters published	Publications in peer-reviewed journals, including original research and commentaries	148
		Books	4
		Book chapters	15
	Citations – journal articles	Citations of journal articles	2461^a^
		% of publications in top 10% most cited worldwide	20.8%^a^
		Average field-weighted citation impact	1.54^a^
		Average number of citations per publication	17.9^a^
	Citations – books and book chapters	Citations of books and book chapters	128^b^
	Altmetrics	Total Altmetric mentions for CRE-DH publications; average per publication	5710; 39 per publication^c^
	Open access	Open access publications	104^a^
	Views	Views of publications	5679^a^
	Grey literature	Reports	48
		Fact sheets	17
	Conferences	Presentations (including plenary presentations, concurrent sessions, skill-building workshops, etc.)	65 (18 of which were at international forums)
	Media/social media	Media articles (for e.g. *The Conversation*, *MJAInsight*)	132
		Views of media articles published in *The Conversation* (*n* = 38) on original site and republished on another site/platform	524,216^d^
		Mentions for all CRE-DH publications (*n* = 147); news, blog, policy, X mentions, Facebook, Wikipedia	News 3488; blog 40; policy 45; X (formerly Twitter) 2116; Facebook 17; Wikipedia 4^c^
		X (formerly Twitter) followers	2877^e^
	Newsletters	No. of newsletters	58^f^
		Reach of newsletters	1283 subscribers, 45% open rate^f^
Capacity strengthening	Collaborations – individuals	Unique authors on publications	194 unique authors^a^
	Collaborations – organizations	Unique organizations represented on publications	57 unique institutions^a^ (46 unique academic and 11 non-academic)
		No. and type of non-academic organizations on represented publications	11 different government departments and hospitals^a^
		Unique international organizations	34^a^
	Disability representation	CRE-DH investigators who identify as having a disability	4
		No. and percentage of publications with at least one chief or associate investigator with disability listed as an author	85 of 147, 58%
	PhDs	PhD students funded by CRE-DH	2
		PhD students affiliated with the CRE-DH	7
		PhD completions	7
	Research positions	Research-related positions funded by the CRE-DH	9
		Researchers affiliated with the CRE-DH	25
	Capacity-strengthening events	Workshops, forums and public seminars facilitated by CRE-DH	11
Policy contribution	Contribution to policy	Submissions to policy and parliamentary processes including inquiries and Royal Commissions	29
		Chief investigator participation in relevant committees	40 unique committees
Economic impact	Leveraged grants, consultancies and fellowships	Research grants and consultancies leveraged	49
		Fellowships leveraged	4, for example, Australian Research Council Future Fellowship
		Value of research grants and consultancies leveraged attributed to CRE-DH	$39,960,798 (sensitivity analysis 26.6–53.2 million) (2024 AUD)
	Increase in lifetime earnings	Increase in lifetime earnings from completed PhDs	$380,522.19 (2024 AUD)
Community benefit		Reduced inequalities in health outcomes between people with and without disability	Increase visibility of issues relating to health inequalities

Data sources are CRE-DH project records, unless otherwise stated: ^a^SciVal, 17 December 2024; ^b^Google Scholar, 17 December 2024; ^c^Altmetric Explorer, 17 December 2024; ^d^*The Conversation*, 6 November 2024; ^e^X, 17 December 2024; ^f^MailChimp, 10 October 2024. Final data sourcing date was 31/12/2024, 1 year after the CRE-DH extension ended

The estimated increase in lifetime earnings associated with PhD completion was put at $54,360 (2024 AUD) per graduate [27].

### Narrative

Qualitative data were collected as part of the CRE-DH evaluation between January and February 2023 [12]. The aim was to understand how diverse stakeholders engaged with the CRE-DH and perceived its impact. Seven interviews were held with senior policy staff across government and disability organizations. These interviewees had had some involvement with the CRE-DH, either as members of the Partner Advisory Group or through accessing workshops and outputs. They included representatives from the Commonwealth Department of Social Services, National Disability Insurance Agency, Commonwealth Department of Health and Aged Care, Human Rights Commission, Australian Institute of Health and Welfare and Disabled People’s Organisations Australia. Organizational and departmental interviews were conducted by H.D., a chief investigator of the CRE-DH. We also interviewed four CRE-DH early career researchers to understand how the Centre had strengthened research capacity. These interviews were conducted by S.Y. who, as an ECR affiliate but not an employee of the CRE-DH, could encourage researchers to speak freely about their experiences.

Interviews were transcribed and initially analysed by H.D. and S.Y. to identify key themes. J.B. then applied a deductive approach to organize the identified themes within the impact domains outlined in the program logic model. This process formed the foundation of the narrative, highlighting key aspects of impact. To do this, J.B. immersed herself in the data, reading and re-reading entries to get a sense of the whole. She then coded the data to each of the domains of impact, writing notes in the process. The coding was cross-checked by H.D. and S.Y. to ensure it aligned with their understanding of the data. Through a process of collaborative writing and revisiting the data, J.B., H.D. and S.Y. refined the analysis and integrated the qualitative insights with other data sources as described below.

### Integration of methods

J.B. and H.D. reviewed and integrated the findings from the three data sources using the five research impact domains as the guiding framework. The process was iterative and interpretive, involving multiple cycles of review, dialogue and reflection with the broader author team to examine alignment and divergence across data sources. This approach provided a form of triangulation, enabling collective interpretation and validation of the findings.

## Results

The retrospective application of FAIT to the CRE-DH identified areas of research impact from 2016 to 2024 in five benefit domains. We present a synthesis of our findings under these domains in Table 2.

### Knowledge advancement

The work of the CRE-DH has highlighted inequalities in health and social determinants faced by Australians with disability, how social determinants impact on their health, and whether policies in this area have been effective. By analysing existing Australian administrative datasets, the CRE-DH provided an evidence base to support its stakeholders in making informed policy decisions about the health and wellbeing of people with disability.

The CRE-DH produced numerous outputs, including 148 peer-reviewed publications, 4 books, 15 book chapters, 48 reports, 17 fact sheets (see Appendix 2 for a full listing), 65 conference presentations and 58 newsletters (see Table 1). Publications have been cited more than 2400 times, with 21% ranking in the top 10% most cited globally.

For research translation, the CRE-DH authored 132 media articles; 38 of these were published in *The Conversation,* a research-based news and analysis media outlet, and garnered more than 524,000 views. Altmetric identified 3488 news feature mentions of CRE-DH publications. Despite these efforts, some non-government stakeholder interviewees reported challenges in integrating the CRE-DH “blue-sky thinking” into practice, and expressed a desire for improved communication of research findings beyond written reports.

Knowledge advancement was stimulated by bringing together a diverse range of stakeholders to learn, share ideas and solve problems. Before the CRE-DH, stakeholders interviewees felt there had been little engagement between the disability and health sectors, academics, policymakers and service providers. Thus, building a diverse stakeholder network became a priority. The establishment of a Partner Advisory Group, which met quarterly, facilitated the setting of research priorities and a two-way information flow amongst diverse constituencies. This group included members from both government and non-government organizations – such as statutory bodies, peak bodies and disability advocacy organizations – thereby strengthening collaborations and directing stakeholders to CRE-DH projects, research and resources. An external stakeholder remarked, “This is a partnership – it helps that we aren’t just working with one university, but [with] many – and this also helps to find other researchers when we need them”.

This collaborative approach resulted in publications comprising 194 individual authors from 57 organizations, including 46 academic institutions and 11 government departments and hospitals (see Table 1). Notably, the contributions of people with lived experience of disability were encouraged, with several serving as members of the Partner Advisory Group and others as expert advisors and valued contributors to the academic research team. Their expertise enhanced the quality and practicality of the CRE-DH’s outputs. An example of this is the Disability and Wellbeing Monitoring Framework, [28] which was the first of its kind to have people with lived experience of disability identifying the most critical social determinants affecting their health.

### Capacity strengthening

The CRE-DH approached capacity strengthening through three main avenues: developing a highly trained workforce of graduate students and ECRs; strengthening the disability research workforce by building research capacity in disability across disciplines (e.g. health economics, epidemiology, health, social sciences and policy); and enhancing capacity in disability policy across sectors (including health, welfare and government statistical agencies).

The CRE-DH provided direct scholarship funding for two PhD students who went on to secure other funding sources. An additional nine PhD students, funded through other mechanisms, were supported by the CRE-DH through networking and training activities. Additionally, the CRE-DH provided funding support for nine ECRs and an additional 25 affiliated researchers, excluding investigators.

ECR interviewees expressed positive feedback regarding the professional development opportunities provided by the CRE-DH. However, the original goal of offering comprehensive mentoring was not fully realized due to team members moving between institutes and roles, changing personal circumstances over the 6 years, and the unexpected death of the staff member leading the mentorship program. As a result, mentoring support was not sustained throughout the CRE-DH’s tenure and its focus was primarily on academic careers rather than industry or government pathways. Although funds were allocated for training and development, not all ECR team members were aware of the available opportunities. As a result, some felt their leadership skills could have been developed more fully or earlier in their involvement with the CRE-DH. Furthermore, due to COVID-19 restrictions the CRE-DH was unable to support its initial ambition of placing ECRs with international partners (e.g. WHO).

The CRE-DH provided research opportunities for scholars with specific methodological expertise (e.g. in economics), but who had not previously focussed on disability research. Many of these researchers have continued in the field, becoming chief investigators on the Centre of Research Excellence in Achieving Health Equity for All People with Disabilities (AHEAD) (see Fig. 1) [29] and securing research fellowships in the disability field. This significantly strengthens disability research capacity.

In total, 11 workshops and public seminars were conducted as capacity-strengthening activities for personnel from multiple sectors (see Appendix 3 for a list). For example, an online 2-day policy forum held in September 2022 considered how health and disability systems could be reformed to improve people with disability’s health outcomes [30]. The forum attracted 32 representatives from 15 organizations, including the Commonwealth Department of Health and Aged Care and Department of Social Services, the National Disability Insurance Agency, the National Disability Insurance Scheme Quality and Safeguards Commission, the Australian Bureau of Statistics, the Australian Institute of Health and Welfare and the Australian Human Rights Commission.

At the request of external stakeholders in the disability sector, the CRE-DH also provided professional development and training webinars. In August 2022, a webinar on using quantitative data for disability research attracted 301 attendees from more than 80 organizations. Training opportunities such as this were highly valued, with one external stakeholder noting, “[The CRE-DH training session]… really helped our junior staff to get their heads around datasets and how to use these”. ECR interviewees valued the opportunity to contribute to and lead such multisectoral events. One commented that “…6 years down the track, I do feel like I have quite a lot of contacts within a lot of different government agencies. Reflecting on that, it does feel like the CRE-DH has enabled me to develop those connections”. Comments such as these highlight the role of the Centre in facilitating long-term professional networks and fostering ongoing collaboration across sectors.

Disability representation was an important component of the Centre’s capacity-strengthening approach. Amongst the 19 CRE-DH investigators, 4 identified as having a disability, including the lead chief investigator. Additionally, 58% of publications had at least one person with disability listed as an author. However, despite the CRE-DH recruiting some staff and students with disability and maintaining a core commitment to working closely with individuals who have lived experience of disability, the Centre did not systematically identify these individuals in its administrative records, resulting in incomplete data.

### Policy contribution

The CRE-DH contributed to policy through 29 submissions to major national strategies and inquiries, including the National Disability Strategy and the Royal Commission into Violence, Abuse, Neglect, and Exploitation of People with Disability [31]. Altmetric identified 45 citations of CRE-DH publications in policy documents, underscoring the relevance of its work to policymakers.

External stakeholders widely acknowledged the CRE-DH’s development of an evidence base as being crucial for their work programs. Government agencies particularly valued the CRE-DH’s timely evidence on pertinent issues, with one external stakeholder noting, “Evidence generated by the CRE-DH can help us to test ideas we have been developing against an evidence base”. Others commented on the comprehensive, integrated and distinctive quality of the research: “The CRE-DH developed a full evidence base that brings all of the data together and disaggregates by sex and is not ableist. And that’s unusual”. Similarly: “The reports come out and I can immediately rely on them. It is a source of truth and one that I can rely on … there’s almost no data capacity in the advocacy sector and we need this”. As a result of this reputation, the WHO used the CRE-DH as a case study of exemplar practice in moving the disability agenda forward in its Global Report on Health Equity for Persons with Disabilities [1].

CRE-DH investigators provided expert input to more than 40 committees, including the COVID-19 Disability Advisory Committee, the WHO Collaborating Centre for Strengthening Rehabilitation Capacity and the Disability Employment Services Reform Taskforce. Their membership of these committees, including by ministerial invitation, facilitated contributions to international, national and state disability agendas through two-way knowledge sharing. As one external stakeholder commented, “I just want to emphasize how valuable the CRE is in keeping those of us in government honest”.

The CRE-DH also responded to emerging community-driven and policy priorities, such as protecting the health of people with disability during the COVID-19 pandemic. Statements of concern with detailed recommendations led to policy action and practice changes in areas such as managing COVID-19 outbreaks across disability residential settings and addressing vaccine hesitancy amongst disability support workers. Expert submissions and witness statements informed the recommendations of the Royal Commission into Violence, Abuse, Neglect, and Exploitation of People with Disability [31], which were endorsed by the Commonwealth Government. These included an ongoing specific advisory committee related to disability and COVID-19, as well as guidelines for responses in disability accommodation settings. The CRE-DH reviewed practice standards and quality indicators to enable providers to respond better to future pandemics, and produced recommendations for ongoing access to personal protective equipment for the disability support workforce. External stakeholders frequently mentioned the CRE-DH’s responsiveness to COVID-19 in their interviews, with one noting that “The CRE-DH’s COVID-19 contributions were vital to informing the response from government. So, there’s absolutely been a very concrete contribution”.

### Economic impact

The CRE-DH research cost totalled $5,423,399 (2024 AUD), a figure that includes direct financial and opportunity costs. From 2016 to 2023, the CRE-DH was able to leverage a further $39,960,798 (2024 AUD) in research, consultancies and fellowship funding. The majority of this was from research grants, including a NHMRC-funded Synergy Grant to improve the mental health outcomes for young people with disability (NHMRC GNT2010290). Most of the consequences from the CRE-DH were of an intermediate nature and had not yet translated into improvements in health and wellbeing for people with disability or cost savings. As such, the only other consequence that could be monetarized was the increased lifetime earnings of PhD graduates supported by the CRE-DH, totalling $380,522 (2024 AUD). Given that most of the impact on outcomes is expected in the future, we did not estimate a return on investment as this would be misleading. The monetarized results of the cost-consequence analysis are available in Table 3, but the true value needs to consider the non-monetarized consequences expressed in Table 2, which are difficult to calculate.

**Table 3:** Cost consequence analysis: costs and monetizable consequences for the CRE-DH

	2024 (AUD)^a^
Costs	
In-kind research costs from chief investigator support	$2,165,871
In-kind research costs from workshop attendance	$39,245
100% of CRE-DH grant	$3,179,038
Total costs	$5,423,399
Consequences	
CRE-DH attributable funding leveraged	$39,960,798 (s.a $26,640,532–53,281,064)
Increased lifetime earnings of PhD students	$380,522
Total consequences	$40,341,320

^a^Converted into 2024 AUD using the Consumer Price Index

*s.a* sensitivity analysis

### Community benefit

Although the CRE-DH successfully raised the visibility of health inequities affecting people with disability, and quantified their impacts, we were unable to measure the Centre’s impact on reducing these inequities. However, this was expected and reasonable given the longer timeframes needed to influence health outcomes for people with disability in a meaningful way. In addition, our capacity-building efforts – such as supporting ECRs and strengthening disability research networks – are likely to contribute to longer-term community benefit by fostering sustained research and advocacy in this area.

## Discussion

By assessing the impact of the CRE-DH using FAIT, our study provides valuable insights and new knowledge on the impacts of this cross-disciplinary research collaboration. The CRE-DH advanced knowledge, strengthened capacity, contributed to policy and generated economic impact. However, the timing of the impact assessment meant it was too soon to report any quantifiable benefit on the health and wellbeing of people with disability, or a possible reduction in health inequities. This issue of lag between research and impact has been highlighted by others [15, 32, 33], along with the enduring challenge of attribution, that is, determining the extent to which observed changes can be linked to the research itself, as opposed to broader contextual or external factors.

The later years of the CRE-DH’s operations coincided with the COVID-19 pandemic. This context is important as it led to the CRE-DH playing an unexpected role in highlighting the vulnerability of people with disability during the pandemic and the need for specific policy actions to protect them. Stakeholder interviews indicated the work was highly impactful in a time of great uncertainty, leading to expedited policy and practice change. This development underscores the importance of research programs having the flexibility and agility to respond to emerging priorities whilst maintaining their broader strategic goals.

Increasingly, researchers are responding to the call of “nothing about us without us” [34] by ensuring that people with disability are engaged in leadership roles in disability research [35, 36]. Despite having investigators with disability on the team, including the lead chief investigator (A.K.), the CRE-DH did not manage to capture sufficient information on disability representation. This was because it was not possible to determine accurately how many people with disability authored manuscripts, were investigators on grants or attended workshops, as these records were not routinely collected or maintained.

Capturing and measuring the representation of people with disability in research remains challenging [35, 37]. Disclosure is often shaped by experiences of stigma and discrimination, as well as by the degree of safety, trust and inclusion fostered within institutional cultures [36]. People may choose not to disclose their disability status, due to concerns about confidentiality or potential bias, leading to an underestimation of representation. In addition to self-identification, it is important to recognize and document the involvement of allies and those with lived experience of disability through close family or caring relationships. These perspectives can provide valuable insight into the relational and structural dimensions of disability, and strengthen the inclusiveness and relevance of disability research. The process of this impact evaluation has strengthened our aspirations for disability representation and leadership in research, including both people with disability and those with lived experience of disability.

What we measure, and how we measure it, reflect what we value. Traditional academic metrics tend to prioritize outcomes that matter most to researchers or institutions, rather than those valued by people with disability themselves. As perceptions of benefit are shaped by who defines and measures them, expectations and intended outcomes should be negotiated and revisited throughout the research process. The key question is not only whether impact occurs, but also what kinds of benefits are being pursued and for whom. Meaningful impact requires listening to and amplifying the voices of people with disability.

### How did FAIT add value?

This study is one of the first to apply FAIT framework within a disability research collaboration. Accordingly, we adapted FAIT to reflect disability-inclusive research principles by measuring the representation and leadership of people with disability across project activities, authorship and governance. This approach moves beyond a focus on research translation and economic returns to include participation, leadership and inclusion as important indicators of impact. By doing so, we broaden how FAIT can be understood and used, and align impact assessment with equity-driven and participatory research approaches. Here we reflect on what we learned through applying FAIT in this context.

First, FAIT’s domains of benefit provided a useful guide for identifying a range of impacts that extend beyond the traditional focus on academic outputs. For example, whilst we had anecdotally considered policy engagement and impact as major achievements of the CRE-DH, FAIT offered a structured approach through which to evaluate these.

Second, although FAIT is intended to be applied prospectively, we applied it retrospectively. Whilst this approach was still beneficial, it presented challenges due to a lack of certain data that could have demonstrated impact more effectively. For example, we did not have records of attendance at all capacity-strengthening events or the number of affiliated PhD completions; these are important metrics both for assessing the impact of capacity-strengthening activities and for contributing to an economic analysis. Another example is the lack of consistent disability inclusion data as discussed earlier.

Third, although FAIT recommends developing a program logic model, which is a useful tool for articulating the pathway to impact, we found that depicting the CRE-DH linearly and failing to place it in context was a limitation. As such, a growing number of researchers are advocating for methods grounded in systems-thinking concepts to address criticisms of linear program logic models [38]. These more iterative system approaches are potentially better suited to large-scale programs of work such as the CRE-DH that actively encourage affiliated membership and activities and embed feedback loops.

Fourth, FAIT categorizes further research investment as a benefit rather than an additional cost or subsequent input. Whilst the $39 million (AUD 2024) of additional funding leveraged from the CRE-DH may be a strong signal of the Centre’s success, these additional funds could alternatively be viewed as an input into ongoing expanded programs of work. We argue that this further investment should, at this stage, be viewed instead as an intermediate outcome. A future evaluation of the CRE-DH may then want to consider this further investment as a cost, and estimate the direct benefits of such an investment on the health outcomes of people with disability.

Finally, defining evaluation boundaries was particularly difficult for the CRE-DH because it was a research collaboration. As such, we struggled to determine the extent to which impact was attributable to the CRE-DH versus the other grants, projects or activities with which our researchers were affiliated.

Fortunately, we have secured the next 5-year iteration of the CRE-DH through a funding grant for the Centre of Research Excellence in Achieving Health Equity for All People with Disabilities (AHEAD, NHMRC #2035278) (see Fig. 1) [29]. Box 1 summarizes our lessons for future cross-disciplinary impact assessments. These lessons are being consolidated into our new initiative to improve its design and implementation, and will allow us to capture our impact more effectively as the work progresses. We anticipate these lessons will have broader applicability to those looking to establish research collaborations and accurately measure their impacts.

Box 1: Recommendations for future cross-disciplinary impact assessments

1. Identify the pathway to impact early, emphasizing research translation throughout the process.
2. Integrate systems-thinking approaches to reflect complexity, interdependencies and nonlinear pathways to impact.
3. Evaluate the overall impact of the collaboration, rather than assessing impact on a project-by-project basis.
4. Invest in ongoing evaluation (not just at the end of the collaboration), using approaches such as developmental evaluation, to inform the formation, functioning and outcomes of the collaboration.
5. Establish processes that will consistently collect and collate detailed data to demonstrate research collaboration impact.
6. Include disability identifiers (as appropriate) for research team, outputs and activities; this includes people with disability and people with lived experience of disability.

Leadership, encouragement and funding from research bodies are essential for understanding and demonstrating the value of research investments [39]. Sustained and dedicated financial and leadership support for evaluation enables systematic assessment of impacts, ongoing learning from successes and challenges and the generation of evidence to inform future research and policy. Without adequate investment and leadership commitment, opportunities to capture impact and strengthen translational pathways are often missed.

### Limitations

Our findings must be considered within the following limitations. As most of the authors were embedded within the CRE-DH, they could be viewed as having a vested interest in presenting the collaboration in a positive light. There is also the potential for survivorship bias [40]: as all the interviewees were people who had continued to work with the CRE-DH, it is possible that those who left the collaboration after a short time may have had a less favourable experience, but their views are not represented in these data. Nor did we record the number of individuals who left the collaboration, further limiting our ability to assess the scope of missing perspectives. As a result, the narratives of longstanding collaborators could reflect recall bias and positivity bias. Additionally, the relatively small number of interviews may not fully capture the diversity of experiences across such a large and cross-disciplinary collaboration.

However, the credibility of our findings was enhanced by several factors: our analysis was supported by triangulation across multiple data sources, and interviews were conducted by two researchers, one of whom was not involved in the initial collaboration. Although they were research affiliates, the lead author (J.B.) and two other authors (S.Y. and S.D.) were not members of the management committee, chief investigators or employed through the CRE-DH. Collaborative interpretation of results with these co-authors further strengthened objectivity. At times, limited data constrained our ability to report on impact domains, which then required a more conservative approach to reporting. In addition, estimates for the lifetime monetary returns from a PhD are based on studies from more than two decades ago, however, more recent evidence suggests that returns to a PhD have been stable over this period [41].

## Conclusions

In this paper, we retrospectively applied FAIT to explore the research impact of a cross-disciplinary research collaboration aimed at improving the health of working age Australians with disability. Whilst cross-disciplinary research endeavours increasingly aim to understand and help reduce inequities, their impact has not often been examined or explored in detail.

The CRE-DH demonstrated significant impact in several domains: advancing knowledge, strengthening capacity, influencing policy and generating economic benefits. Although quantifiable community benefit data are not yet available, these achievements underscore the value of cross-disciplinary collaborations in addressing complex societal issues.

Our assessment was sometimes limited by insufficient data collection on critical issues, such as disability representation. This highlights the need for more comprehensive data collection strategies to be developed at the outset of collaborations, and for collective thinking about optimal pathways to impact in future research endeavours. The lessons we learned from this evaluation have been instrumental in informing the establishment of a new research collaboration, AHEAD. They have improved the design, implementation and evaluation of this initiative, and our aim is to enhance its overall impact.

Our recommendations for using FAIT in research collaborations (Box 1) are intended to support the broader goals of these initiatives. By adopting these recommendations, other research collaborations will be better able to assess and maximize the impact of their work, ultimately contributing to increased community benefit.

## Supplementary Information

Supplementary material 1.

## Data Availability

The datasets used and/or analysed during this study will be available from the corresponding author on reasonable request after the publishing of the results.
