# Supplementary material for: Impact assessment of a national research collaboration improving health outcomes for working-age Australians with disability
Source: Health Res Policy Syst. 2026 Feb 17;24:27. doi: 10.1186/s12961-026-01448-7 (PMC13015006; doi:10.1186/s12961-026-01448-7)
Supplement: Supplementary file 1 — Supplementary material 1. [file 12961_2026_1448_MOESM1_ESM.docx]

**Appendix 1: Full Listing of CRE-DH Chief and Associate Investigators**

## **Chief Investigators**

| **Name** | **Affiliation** |
| --- | --- |
| Professor Anne Kavanagh | University of Melbourne |
| Professor Emerita Gwynnyth Llewellyn | University of Sydney |
| Professor Eric Emerson | University of Sydney |
| Professor Dennis Petrie | Monash University |
| Professor Helen Dickinson | UNSW Canberra |
| Professor Hannah Badland | RMIT University |
| Professor Peter Butterworth | Australian National University |
| Professor Emeritus Roger Stancliffe | University of Sydney |
| Professor Gemma Carey | University of Melbourne |
| Associate Professor Allison Milner | University of Melbourne |

## **Associate Investigators**

| **Name** | **Affiliation** |
| --- | --- |
| Professor Maria Alarcos Cieza | World Health Organization |
| Professor Karl Atkin | University of York |
| Dr Gloria Krahn | Oregon State University |
| Professor Thomas Shakespeare | London School of Hygiene & Tropical Medicine |
| Professor Luis Salvador Carulla | Australian National University |
| Professor Rebecca Bentley | University of Melbourne |
| Associate Professor Melanie Davern | RMIT University |
| Professor John Gilroy | University of Sydney |
| Professor Jen Smith-Merry | University of Melbourne |

**Appendix 2: CRE-DH Publications, Books, Book Chapters, Reports and Fact Sheets, by Year Published**

## **2.1 Publications**

### **2016**

1. Emerson E, Krnjacki L, Llewellyn G, Vaughan C, Kavanagh A. Perceptions of safety and exposure to violence in public places among working age adults with disabilities or long-term health conditions in the UK: cross sectional study. Public Health. 2016;135:91-6. doi:10.1016/j.puhe.2015.10.036.
2. Gilroy J, Emerson E. Australian indigenous children with low cognitive ability: Family and cultural participation. Res Dev Disabil. 2016;56:117-27. doi:10.1016/j.ridd.2016.05.011.
3. Kavanagh AM, Aitken Z, Baker E, LaMontagne AD, Milner A, Bentley R. Housing tenure and affordability and mental health following disability acquisition in adulthood. Soc Sci Med. 2016;151:225-32. doi:10.1016/j.socscimed.2016.01.010.
4. Kavanagh AM, Aitken Z, Emerson E, Sahabandu S, Milner A, Bentley R, LaMontagne AD, Pirkis J, Studdert D. Inequalities in socio-economic characteristics and health and wellbeing of men with and without disabilities: a cross-sectional analysis of the baseline wave of the Australian Longitudinal Study on Male Health. BMC Public Health. 2016;16:9. doi:10.1186/s12889-016-3700-y.
5. Krnjacki L, Emerson E, Llewellyn G, Kavanagh AM. Prevalence and risk of violence against people with and without disabilities: findings from an Australian population-based study. Aust NZ J Publ Health. 2016;40(1):16-21. doi:10.1111/1753-6405.12498.
6. Williams I, Dickinson H. Going It Alone or Playing to the Crowd? A Critique of Individual Budgets and the Personalisation of Health Care in the English National Health Service. Aust J Public Adm. 2016;75(2):149-58. doi:10.1111/1467-8500.12155.

### **2017**

1. Aitken Z, Krnjacki L, Kavanagh AM, LaMontagne AD, Milner A. Does social support modify the effect of disability acquisition on mental health? A longitudinal study of Australian adults. Soc Psychiatry Psychiatr Epidemiol. 2017;52(10):1247-55. doi:10.1007/s00127-017-1418-5.
2. Aitken Z, Simpson JA, Bentley R, Kavanagh AM. Disability acquisition and mental health: effect modification by demographic and socioeconomic characteristics using data from an Australian longitudinal study. BMJ Open. 2017;7(9):11. doi:10.1136/bmjopen-2017-016953.
3. Carey G, Dickinson H. A longitudinal study of the implementation experiences of the Australian National Disability Insurance Scheme: investigating transformative policy change. BMC Health Serv Res. 2017;17:6. doi:10.1186/s12913-017-2522-7.
4. Carey G, Malbon E, Reeders D, Kavanagh A, Llewellyn G. Redressing or entrenching social and health inequities through policy implementation? Examining personalised budgets through the Australian National Disability Insurance Scheme. Int J Equity Health. 2017;16:12. doi:10.1186/s12939-017-0682-z.
5. Dickinson H. Individual funding systems: What works?. Evidence Base: A journal of evidence reviews in key policy areas. 2017;1(3):1-8. doi:10.21307/eb-2017-003
6. Dickinson H, Carey G. Managing care integration during the implementation of large-scale reforms The case of the Australian National Disability Insurance Scheme. J Integr Care. 2017;25(1):6-16. doi:10.1108/jica-07-2016-0026.
7. Hindmarsh G, Llewellyn G, Emerson E. The Social-Emotional Well-Being of Children of Mothers with Intellectual Impairment: A Population-Based Analysis. J Appl Res Intellect Disabil. 2017;30(3):469-81. doi:10.1111/jar.12306.
8. Milner A, Aitken Z, Kavanagh A, LaMontagne AD, Petrie D. Status inconsistency and mental health: A random effects and instrumental variables analysis using 14 annual waves of cohort data. Soc Sci Med. 2017;189:129-37. doi:10.1016/j.socscimed.2017.08.001.
9. Milner A, King TL, LaMontagne AD, Aitken Z, Petrie D, Kavanagh AM. Underemployment and its impacts on mental health among those with disabilities: evidence from the HILDA cohort. J Epidemiol Community Health. 2017;71(12):1198-202. doi:10.1136/jech-2017-209800.

### **2018**

1. Aitken Z, Simpson JA, Gurrin L, Bentley R, Kavanagh AM. Do material, psychosocial and behavioural factors mediate the relationship between disability acquisition and mental health? A sequential causal mediation analysis. Int J Epidemiol. 2018;47(3):829-40. doi:10.1093/ije/dyx277.
2. Carey G, Dickinson H, Malbon E, Reeders D. The Vexed Question of Market Stewardship in the Public Sector: Examining Equity and the Social Contract through the Australian National Disability Insurance Scheme. Soc Policy Adm. 2018;52(1):387-407. doi:10.1111/spol.12321.
3. Clifton S, Llewellyn G, Shakespeare T. Quadriplegia, virtue theory, and flourishing: a qualitative study drawing on self-narratives. Disabil Soc. 2018;33(1):20-38. doi:10.1080/09687599.2017.1379951.
4. Emerson E. Smoking among adults with and without disabilities in the UK. J Public Health. 2018;40(4):E502-E9. doi:10.1093/pubmed/fdy062.
5. Kavanagh A, Priest N, Emerson E, Milner A, King T. Gender, parental education, and experiences of bullying victimization by Australian adolescents with and without a disability. Child Care Health Dev. 2018;44(2):332-41. doi:10.1111/cch.12545.
6. King T, Aitken Z, Milner A, Emerson E, Priest N, Karahalios A, Kavanagh A, Blakely T. To what extent is the association between disability and mental health in adolescents mediated by bullying? A causal mediation analysis. Int J Epidemiol. 2018;47(5):1402-13. doi:10.1093/ije/dyy154.
7. Krnjacki L, Priest N, Aitken Z, Emerson E, Llewellyn G, King T, Kavanagh A. Disability-based discrimination and health: findings from an Australian-based population study. Aust NZ J Publ Health. 2018;42(2):172-4. doi:10.1111/1753-6405.12735.
8. Malbon E, Carey G, Dickinson H. Accountability in Public Service Quasi-markets: The Case of the Australian National Disability Insurance Scheme. Aust J Public Adm. 2018;77(3):468-81. doi:10.1111/1467-8500.12246.
9. Milner A, Aitken Z, Kavanagh A, LaMontagne AD, Pega F, Petrie D. Combining fixed effects and instrumental variable approaches for estimating the effect of psychosocial job quality on mental health: evidence from 13 waves of a nationally representative cohort study. J Public Health. 2018;40(2):426-34. doi:10.1093/pubmed/fdx070.
10. Milner A, Law PCF, Mann C, Cooper T, Witt K, LaMontagne AD. A smart-phone intervention to address mental health stigma in the construction industry: A two-arm randomised controlled trial. SSM-Popul Health. 2018;4:164-8. doi:10.1016/j.ssmph.2017.12.007.
11. Milner A, Taouk Y, Disney G, Aitken Z, Rachele J, Kavanagh A. Employment predictors of exit from work among workers with disabilities: A survival analysis from the household income labour dynamics in Australia survey. PLoS One. 2018;13(12):14. doi:10.1371/journal.pone.0208334.
12. Milner A, Witt K, LaMontagne AD, Niedhammer I. Psychosocial job stressors and suicidality: a meta-analysis and systematic review. Occup Environ Med. 2018;75(4):245-53. doi:10.1136/oemed-2017-104531.
13. Needham C, Dickinson H. 'Any one of us could be among that number': Comparing the Policy Narratives for Individualized Disability Funding in Australia and England. Soc Policy Adm. 2018;52(3):731-49. doi:10.1111/spol.12320.
14. Niedhammer I, Milner A, Witt K, Klingelschmidt J, Khireddine-Medouni I, Alexopoulos EC, Toivanen S, Chastang JF, LaMontagne AD. Response to letter to the editor from Dr Rahman Shiri: The challenging topic of suicide across occupational groups. Scand J Work Environ Health. 2018;44(1):108-10. doi:10.5271/sjweh.3698.
15. Patton KA, Ware R, McPherson L, Emerson E, Lennox N. Parent-Related Stress of Male and Female Carers of Adolescents with Intellectual Disabilities and Carers of Children within the General Population: A Cross-Sectional Comparison. J Appl Res Intellect Disabil. 2018;31(1):51-61. doi:10.1111/jar.12292.

### **2019**

1. Aitken Z, Baker E, Badland H, Mason K, Bentley R, Beer A, Kavanagh AM. Precariously placed: housing affordability, quality and satisfaction of Australians with disabilities. Disabil Soc. 2019;34(1):121-42. doi:10.1080/09687599.2018.1521333.
2. Arciuli J, Emerson E, Llewellyn G. Adolescents' Self-Report of School Satisfaction: The Interaction Between Disability and Gender. School Psychol. 2019;34(2):148-58. doi:10.1037/spq0000275.
3. Bollier AM, King T, Shakespeare T, Hocking J, Kavanagh A. Sexual Functioning in Men With and Without Disabilities: Findings From a Representative Sample of Australian Men. J Sex Med. 2019;16(11):1749-57. doi:10.1016/j.jsxm.2019.07.021.
4. Carey G, Malbon ER, Weier M, Dickinson H, Duff G. Making markets work for disability services: The question of price setting. Health Soc Care Community. 2019;27(5):E716-E23. doi:10.1111/hsc.12780.
5. Emerson E, King T, Llewellyn G, Milner A, Aitken Z, Arciuli J, Kavanagh A. Emotional difficulties and self-harm among British adolescents with and without disabilities: Cross sectional study. Disabil Health J. 2019;12(4):581-7. doi:10.1016/j.dhjo.2019.04.007.
6. King TL, Milner A, Aitken Z, Karahalios A, Emerson E, Kavanagh AM. Mental health of adolescents: variations by borderline intellectual functioning and disability. Eur Child Adolesc Psych. 2019;28(9):1231-40. doi:10.1007/s00787-019-01278-9.
7. King TL, Shields M, Shakespeare T, Milner A, Kavanagh A. An intersectional approach to understandings of mental health inequalities among men with disability. SSM-Popul Health. 2019;9:8. doi:10.1016/j.ssmph.2019.100464.
8. Malbon E, Alexander D, Carey G, Reeders D, Green C, Dickinson H, Kavanagh A. Adapting to a marketised system: Network analysis of a personalisation scheme in early implementation. Health Soc Care Community. 2019;27(1):191-8. doi:10.1111/hsc.12639.
9. Malbon E, Carey G, Meltzer A. Personalisation schemes in social care: are they growing social and health inequalities? BMC Public Health. 2019;19:12. doi:10.1186/s12889-019-7168-4.
10. Mellifont D, Smith-Merry J, Dickinson H, Llewellyn G, Clifton S, Ragen J, Raffaele M, Williamson P. The ableism elephant in the academy: a study examining academia as informed by Australian scholars with lived experience. Disabil Soc. 2019;34(7-8):1180-99. doi:10.1080/09687599.2019.1602510.
11. Milner A, Aitken Z, Law PCF, LaMontagne AD, Mann C, Cooper T, Witt K. The relationship between an electronic mental health stigma campaign and suicidal thoughts and behaviours: a two-arm randomized controlled trial in the Australian construction industry. Health Promot Int. 2020;35(3):478-85. doi:10.1093/heapro/daz034.
12. Milner A, Bollier AM, Emerson E, Kavanagh A. The relationship between disability and suicide: prospective evidence from the Ten to Men cohort. J Public Health. 2019;41(4):707-13. doi:10.1093/pubmed/fdy197.
13. Milner A, King TL, Kavanagh A. The mental health impacts of health and human service work: Longitudinal evidence about differential exposure and susceptibility using 16 waves of cohort data. Prev Med Rep. 2019;14:7. doi:10.1016/j.pmedr.2019.100826.
14. Milner A, Petrie D, LaMontagne AD, Butterworth P. Do psychosocial job stressors influence mental health service use? Evidence from an Australian cohort. Occup Environ Med. 2019;76(5):295-301. doi:10.1136/oemed-2018-105440.
15. Milner A, Shields M, King T, Aitken Z, LaMontagne A, Kavanagh AM. Disabling working environments and mental health: A commentary. Disabil Health J. 2019;12(4):537-41. doi:10.1016/j.dhjo.2019.06.002.
16. Nevile A, Malbon E, Kay A, Carey G. The implementation of complex social policy: Institutional layering and unintended consequences in the National Disability Insurance Scheme. Aust J Public Adm. 2019;78(4):562-76. doi:10.1111/1467-8500.12380.
17. Olney S, Dickinson H. Australia's New National Disability Insurance Scheme: Implications for Policy and Practice. Policy Des Pract. 2019;2(3):275-90. doi:10.1080/25741292.2019.1586083.
18. Xu TX, Stancliffe RJ. An evaluation of employment outcomes achieved by transition to work service providers in Sydney, Australia. J Intellect Dev Dis. 2019;44(1):51-63. doi:10.3109/13668250.2017.1310809.
19. Zhou QS, Llewellyn G, Stancliffe R, Fortune N. Working-age people with disability and labour force participation: Geographic variations in Australia. Aust J Soc Iss. 2019;54(3):323-40. doi:10.1002/ajs4.75.

### **2020**

1. Aitken Z, Simpson JA, Bentley R, Milner A, LaMontagne AD, Kavanagh AM. Does the effect of disability acquisition on mental health differ by employment characteristics? A longitudinal fixed-effects analysis. Soc Psychiatry Psychiatr Epidemiol. 2020;55(8):1031-9. doi:10.1007/s00127-019-01783-x.
2. Bollier AM, King T, Austin SB, Shakespeare T, Spittal M, Kavanagh A. Does sexual orientation vary between disabled and non-disabled men? findings from a population-based study of men in Australia. Disabil Soc. 2020;35(10):1641-59. doi:10.1080/09687599.2019.1689925.
3. Brotherton M, Stancliffe RJ, Wilson NJ, O'Loughlin K. Australians with intellectual disability share their experiences of retirement from mainstream employment. J Appl Res Intellect Disabil. 2020;33(5):905-16. doi:10.1111/jar.12712.
4. Carey G. The National Disability Insurance Scheme andCOVID-19: a collision course. Med J Aust. 2020;213(3):2. doi:10.5694/mja2.50690.
5. Carey G, Dickinson H, Malbon E, Weier M, Duff G. Burdensome Administration and Its Risks: Competing Logics in Policy Implementation. Adm Soc. 2020;52(9):1362-81. doi:10.1177/0095399720908666.
6. Clifton S, Fortune N, Llewellyn G, Stancliffe RJ, Williamson P. Lived Expertise and the Development of a Framework for Tracking the Social Determinants, Health, and Wellbeing of Australians with Disability. Scand J Disabil Res. 2020;22(1):137-46. doi:10.16993/sjdr.688.
7. Courtenay K. Covid-19: challenges for people with intellectual disability. BMJ-British Medical Journal. 2020;369:1. doi:10.1136/bmj.m1609.
8. Curryer B, Stancliffe RJ, Wiese MY, Dew A. The experience of mothers supporting self-determination of adult sons and daughters with intellectual disability. J Appl Res Intellect Disabil. 2020;33(3):373-85. doi:10.1111/jar.12680.
9. Devine A, Vaughan C, Kavanagh A. If I had stable housing I would be a bit more receptive to having a job. Factors influencing the effectiveness of Disability Employment Services reform. Work. 2020;65(4):775-87. doi:10.3233/wor-203130.
10. Devine A, Vaughan C, Kavanagh A, Dickinson H, Byars S, Dimov S, Gye B, Brophy L. 'I'm proud of how far I've come. I'm just ready to work': mental health recovery narratives within the context of Australia's Disability Employment Services. BMC Public Health. 2020;20(1):18. doi:10.1186/s12889-020-8452-z.
11. Dickinson H, Carey G, Kavanagh AM. Personalisation and pandemic: an unforeseen collision course? Disabil Soc. 2020;35(6):1012-7. doi:10.1080/09687599.2020.1772201.
12. Disney G, Gurrin L, Aitken Z, Emerson E, Milner A, Kavanagh A, Petrie D. Hierarchical Models for International Comparisons Smoking, Disability, and Social Inequality in 21 European Countries. Epidemiology. 2020;31(2):282-9. doi:10.1097/ede.0000000000001154.
13. Emerson E, Aitken Z, Krnjacki L, Vaughan C, Llewellyn G, Kavanagh A. Torturing inadequate data to generate 'results'? J Public Health. 2020;42(4):E575-E7. doi:10.1093/pubmed/fdz161.
14. Emerson E, Fortune N, Aitken Z, Hatton C, Stancliffe R, Llewellyn G. The wellbeing of working-age adults with and without disability in the UK: Associations with age, gender, ethnicity, partnership status, educational attainment and employment status. Disabil Health J. 2020;13(3):7. doi:10.1016/j.dhjo.2020.100889.
15. Emerson E, Milner A, Aitken Z, Vaughan C, Llewellyn G, Kavanagh AM. Exposure to discrimination and subsequent changes in self-rated health: prospective evidence from the UK's Life Opportunities Survey. Public Health. 2020;185:176-81. doi:10.1016/j.puhe.2020.04.038.
16. Fortune N, Singh A, Badland H, Stancliffe RJ, Llewellyn G. Area-Level Associations between Built Environment Characteristics and Disability Prevalence in Australia: An Ecological Analysis. Int J Environ Res Public Health. 2020;17(21):15. doi:10.3390/ijerph17217844.
17. Karahalios A, Pega F, Aitken Z, Milner A, Simpson JA, Kavanagh AM. The cumulative effect of living with disability on mental health in working-age adults: an analysis using marginal structural models. Soc Psychiatry Psychiatr Epidemiol. 2020;55(3):309-18. doi:10.1007/s00127-019-01688-9.
18. Kavanagh A. Disability and public health research in Australia. Aust NZ J Publ Health. 2020;44(4):262-4. doi:10.1111/1753-6405.13003.
19. King TL, Shields M, Milner A, Vaughan C, Shakespeare T, Currier D, Kavanagh AM. Conformity to Masculine Norms: Differences Between Men With an Without a Disability. Psychol Men Masculinities. 2020;21(3):380-91. doi:10.1037/men0000236.
20. Martínez-Leal R, Folch A, Munir K, Novell R, Salvador-Carulla L. The Girona declaration on borderline intellectual functioning. Lancet Psychiatry. 2020;1;7(3):e8. doi: 10.1016/S2215-0366(20)30001-8
21. McAllister A. Mental illnesses are not an 'ideal type' of disability for disability income support: Perceptions of policymakers in Australia and Canada. Scand J Public Health. 2020;48(4):452-9. doi:10.1177/1403494818816903.
22. McCausland D, Stancliffe RJ, McCallion P, McCarron M. Longitudinal use and factors associated with public transport and other travel options for older people with an intellectual disability in Ireland. J Appl Res Intellect Disabil. 2020;33(3):442-56. doi:10.1111/jar.12686.
23. Meltzer A. Public health crises and the need for accessible information. Med J Aust. 2020;213(10):478-+. doi:10.5694/mja2.50827.
24. Milner A, Aitken Z, Byars S, Butterworth P, Kavanagh A. Do gender and psychosocial job stressors modify the relationship between disability and sickness absence: An investigation using 12 waves of a longitudinal cohort. Scand J Work Environ Health. 2020;46(3):302-10. doi:10.5271/sjweh.3865.
25. Milner A, Disney G, Byars S, King TL, Kavanagh AM, Aitken Z. The effect of gender on mental health service use: an examination of mediation through material, social and health-related pathways. Soc Psychiatry Psychiatr Epidemiol. 2020;55(10):1311-21. doi:10.1007/s00127-020-01844-6.
26. Rachele JN, Disney G, Milner A, Emerson E, Krnjacki L, Kavanagh AM. Violence and Mental Health: Does Disability Make a Difference? Epidemiology. 2020;31(1):E6-E7. doi:10.1097/ede.0000000000001119.
27. Reavley NJ, Morgan AJ, Petrie D, Jorm AF. Does mental health-related discrimination predict health service use 2 years later? Findings from an Australian national survey. Soc Psychiatry Psychiatr Epidemiol. 2020;55(2):197-204. doi:10.1007/s00127-019-01762-2.
28. Savage A, McConnell D, Emerson E, Llewellyn G. The Subjective Well-Being of Adolescent Canadians with Disabilities. J Child Fam Stud. 2020;29(12):3381-97. doi:10.1007/s10826-020-01794-2.

### **2021**

1. Aitken Z, Simpson JA, Bentley R, Kavanagh AM. How much of the effect of disability acquisition on mental health is mediated through employment and income? A causal mediation analysis quantifying interventional indirect effects using data from four waves of an Australian cohort study. BMJ Open. 2021;11(11):10. doi:10.1136/bmjopen-2021-055176.
2. Almeda N, García-Alonso C, Salvador-Carulla L. Mental health planning at a very early stage of the COVID-19 crisis: a systematic review of online international strategies and recommendations. BMC Psychiatry. 2021;21(1):15. doi:10.1186/s12888-020-03015-y.
3. Badji S, Badland H, Rachele JN, Petrie D. Public transport availability and healthcare use for Australian adults aged 18-60 years, with and without disabilities. J Transp Health. 2021;20:8. doi:10.1016/j.jth.2020.101001.
4. Bailie J, Laycock A, Matthews V, Bailie RS. Increasing health assessments for people living with an intellectual disability: lessons from experience of Indigenous-specific health assessments. Med J Aust. 2021;215(1):16-18e1. doi:10.5694/mja2.51124.
5. Butterworth P, de New SC, Schilling C, Saxby K, Petrie D, Wong C. Dynamics of Mental Health and Healthcare Use among Children and Young Adults. Aust Econ Rev. 2021;54(1):130-46. doi:10.1111/1467-8462.12413.
6. Carey G, Malbon E, Blackwell J. Administering inequality? The National Disability Insurance Scheme and administrative burdens on individuals. Aust J Public Adm. 2021;80(4):854-72. doi:10.1111/1467-8500.12508.
7. Clifton S. Disability and the Complexity of Choice in the Ethics of Abortion and Voluntary Euthanasia. J Med Philos. 2021;46(4):431-50. doi:10.1093/jmp/jhab008.
8. Devine A, Dickinson H, Brophy L, Kavanagh A, Vaughan C. 'I don't think they trust the choices I will make.' - Narrative analysis of choice and control for people with psychosocial disability within reform of the Australian Disability Employment Services program. Public Manag Rev. 2021;23(1):10-30. doi:10.1080/14719037.2019.1648700.
9. Devine A, Shields M, Dimov S, Dickinson H, Vaughan C, Bentley R, LaMontagne AD, Kavanagh A. Australia's Disability Employment Services Program: Participant Perspectives on Factors Influencing Access to Work. Int J Environ Res Public Health. 2021;18(21):20. doi:10.3390/ijerph182111485.
10. Emerson E, Fortune N, Llewellyn G, Stancliffe R. Loneliness, social support, social isolation and wellbeing among working age adults with and without disability: Cross-sectional study. Disabil Health J. 2021;14(1):7. doi:10.1016/j.dhjo.2020.100965.
11. Emerson E, Llewellyn G. The exposure of children with and without disabilities to violent parental discipline: Cross-sectional surveys in 17 middle- and low-income countries. Child Abuse Negl. 2021;111:9. doi:10.1016/j.chiabu.2020.104773.
12. Emerson E, Llewellyn G. Youth with disabilities are more likely than their peers to engage in hazardous child labour. Child Care Health Dev. 2021;47(1):119-27. doi:10.1111/cch.12820.
13. Emerson E, Milner A, Aitken Z, Krnjacki L, Vaughan C, Llewellyn G, Kavanagh A. Overt acts of perceived discrimination reported by British working-age adults with and without disability. J Public Health. 2021;43(1):E16-E23. doi:10.1093/pubmed/fdz093.
14. Emerson E, Stancliffe R, Fortune N, Llewellyn G. Disability, Loneliness and Health in the UK: cross-sectional survey. Eur J Public Health. 2021;31(3):533-8. doi:10.1093/eurpub/ckab018.
15. Emerson E, Stancliffe R, Hatton C, Llewellyn G, King T, Totsika V, Aitken Z, Kavanagh A. The impact of disability on employment and financial security following the outbreak of the 2020 COVID-19 pandemic in the UK. J Public Health. 2021;43(3):472-8. doi:10.1093/pubmed/fdaa270.
16. Emerson E, Totsika V, Aitken Z, King T, Hastings RP, Hatton C, Stancliffe RJ, Llewellyn G, Kavanagh A. Vaccine hesitancy among working-age adults with/without disability in the UK. Public Health. 2021;200:106-8. doi:10.1016/j.puhe.2021.09.019.
17. Fortune N, Madden RH, Clifton S. Health and Access to Health Services for People with Disability in Australia: Data and Data Gaps. Int J Environ Res Public Health. 2021;18(21):15. doi:10.3390/ijerph182111705.
18. Griffiths D, Sheehan L, van Vreden C, Petrie D, Grant G, Whiteford P, Sim MR, Collie A. The Impact of Work Loss on Mental and Physical Health During the COVID-19 Pandemic: Baseline Findings from a Prospective Cohort Study. J Occup Rehabil. 2021;31(3):455-62. doi:10.1007/s10926-021-09958-7.
19. Gupta A, Kavanagh A, Disney G. The Impact of and Government Planning and Responses to Pandemics for People with Disability: A Rapid Review. Int J Environ Res Public Health. 2021;18(12):9. doi:10.3390/ijerph18126505.
20. Imms C, Reddihough D, Shepherd DA, Kavanagh A. Social Outcomes of School Leavers With Cerebral Palsy Living in Victoria. Front Neurol. 2021;12:15. doi:10.3389/fneur.2021.753921.
21. Kavanagh A, Dickinson H, Carey G, Llewellyn G, Emerson E, Disney G, Hatton C. Improving health care for disabled people in COVID-19 and beyond: Lessons from Australia and England. Disabil Health J. 2021;14(2):7. doi:10.1016/j.dhjo.2020.101050.
22. King TL. Young carers in the COVID-19 pandemic: risks for mental health Comment. Lancet Reg Health-W Pac. 2021;16:2. doi:10.1016/j.lanwpc.2021.100307.
23. Lukersmith S, Taylor J, Salvador-Carulla L. Vagueness and Ambiguity in Communication of Case Management: A Content Analysis in the Australian National Disability Insurance Scheme. Int J Integr Care. 2021;21(1):13. doi:10.5334/ijic.5590.
24. Maxfield M, Cooper MS, Kavanagh A, Devine A, Atkinson LG. On the outside looking in: a phenomenological study of the lived experience of Australian adults with a disorder of the corpus callosum. Orphanet J Rare Dis. 2021;16(1):13. doi:10.1186/s13023-021-02140-5.
25. Meltzer A, Dickinson H, Malbon E, Carey G. Why is lived experience important for market stewardship? A proposed framework for why and how lived experience should be included in stewarding disability markets. Evid Polciy. 2021;17(2):335-47. doi:10.1332/174426421x16142714946996.
26. Meltzer A, Dowse L, Smith L, Dew A. A framework for interconnected benefits of peer support for family members of people with disability. Aust J Soc Iss. 2021;56(4):579-95. doi:10.1002/ajs4.130.
27. Norman R, Robinson S, Dickinson H, Williams I, Meschcheriakova E, Manipis K, Anstey M. Public preferences for allocation ventilators in an intensive care unit: A discrete choice experiment. The Patient-Patient-Centered Outcomes Research. 2021 May;14:319-30. https://doi.org/10.1007/s40271-021-00498-z
28. Rachele JN, Wang JC, Wijnands JS, Zhao HF, Bentley R, Stevenson M. Using machine learning to examine associations between the built environment and physical function: A feasibility study. Health Place. 2021;70:6. doi:10.1016/j.healthplace.2021.102601.
29. Romero-Lopez-Alberca C, Alonso-Trujillo F, Almenara-Abellan JL, Salinas-Perez JA, Gutierrez-Colosia MR, Gonzalez-Caballero JL, Pulido SP, Salvador-Carulla L. A Semiautomated Classification System for Producing Service Directories in Social and Health Care (DESDE-AND): Maturity Assessment Study. J Med Internet Res. 2021;23(3):15. doi:10.2196/24930.
30. Shields M, Dimov S, Kavanagh A, Milner A, Spittal MJ, King TL. How do employment conditions and psychosocial workplace exposures impact the mental health of young workers? A systematic review. Soc Psychiatry Psychiatr Epidemiol. 2021;56(7):1147-60. doi:10.1007/s00127-021-02077-x.
31. Weld-Blundell I, Shields M, Devine A, Dickinson H, Kavanagh A, Marck C. Vocational Interventions to Improve Employment Participation of People with Psychosocial Disability, Autism and/or Intellectual Disability: A Systematic Review. Int J Environ Res Public Health. 2021;18(22):33. doi:10.3390/ijerph182212083.
32. Yates S, Dickinson H. Navigating Complexity in a Global Pandemic: The Effects of COVID-19 on Children and Young People with Disability and Their Families in Australia. Public Adm Rev. 2021;81(6):1192-6. doi:10.1111/puar.13352.
33. Yates S, Dickinson H, Smith C, Tani M. Flexibility in individual funding schemes: How well did Australia's National Disability Insurance Scheme support remote learning for students with disability during COVID-19? Soc Policy Adm. 2021;55(5):906-20. doi:10.1111/spol.12670.

### **2022**

1. Aitken Z, Bishop GM, Disney G, Emerson E, Kavanagh AM. Disability-related inequalities in health and well-being are mediated by barriers to participation faced by people with disability. A causal mediation analysis. Soc Sci Med. 2022;315:8. doi:10.1016/j.socscimed.2022.115500.
2. Bailie J, Fortune N, Gordon J, Madden RC, Llewellyn G. Making everyone count: it is time to improve the visibility of people with disability in primary care. Med J Aust. 2022;217(4):173-5. doi:10.5694/mja2.51650.
3. Bailie J, Matthews V, Bailie R, Villeneuve M, Longman J. Exposure to risk and experiences of river flooding for people with disability and carers in rural Australia: a cross-sectional survey. BMJ Open. 2022;12(8):10. doi:10.1136/bmjopen-2021-056210.
4. Cunningham R, Milner A, Gibb S, Rijnberg, Disney G, Kavanagh AM. Gendered experiences of unemployment, suicide and self-harm: a population-level record linkage study. Psychol Med. 2022;52(16):4067-75. doi:10.1017/s0033291721000994.
5. Devine A, Dickinson H, Rangi M, Huska M, Disney G, Yang Y, Barney J, Kavanagh A, Bonyhady B, Deane K, McAllister A. 'Nearly gave up on it to be honest': Utilisation of individualised budgets by people with psychosocial disability within Australia's National Disability Insurance Scheme. Soc Policy Adm. 2022;56(7):1056-73. doi:10.1111/spol.12838.
6. Dickinson H, Carey G, Malbon E, Gilchrist D, Chand S, Kavanagh A, Alexander D. Should We Change the Way We Think About Market Performance When It Comes to Quasi-Markets? A New Framework for Evaluating Public Service Markets. Public Adm Rev. 2022;82(5):897-901. doi:10.1111/puar.13392.
7. Doyle C, Yates S, Hargrave J. Reflecting on the Value of Community Researchers in Criminal Justice Research Projects. Soc Sci-Basel. 2022;11(4):14. doi:10.3390/socsci11040166.
8. Emerson E, Aitken Z, King T, Arciuli J, Llewellyn G, Kavanagh AM. The association between disability and risk of exposure to peer cyber victimisation is moderated by gender: Cross-sectional survey. Disabil Health J. 2022;15(1):4. doi:10.1016/j.dhjo.2021.101170.
9. Emerson E, Aitken Z, Totsika V, King T, Stancliffe RJ, Hatton C, Llewellyn G, Hastings RP, Kavanagh A. The impact of the COVID pandemic on working age adults with disability: Meta-analysis of evidence from four national surveys. Health Soc Care Community. 2022;30(6):E4758-E69. doi:10.1111/hsc.13882.
10. Fortune N, Bailie J, Llewellyn G. The need for improved Australian data on social determinants of health inequities. Med J Aust. 2022;217(6):1. doi:10.5694/mja2.51698.
11. Fortune N, Curryer B, Badland H, Smith-Merry J, Devine A, Stancliffe RJ, Emerson E, Llewellyn G. Do Area-Level Environmental Factors Influence Employment for People with Disability? A Scoping Review. Int J Environ Res Public Health. 2022;19(15):16. doi:10.3390/ijerph19159082.
12. Gemma C, Malbon E, Weier M, Duff G. Balancing stability and change: Lessons on policy responsiveness and turbulence in the disability care sector. Health Soc Care Community. 2022;30(4):1307-14. doi:10.1111/hsc.13454.
13. Green C, Dickinson H, Carey G, Joyce A. Barriers to policy action on social determinants of health for people with disability in Australia. Disabil Soc. 2022;37(2):206-30. doi:10.1080/09687599.2020.1815523.
14. Griffiths D, Sheehan L, Petrie D, van Vreden C, Whiteford P, Collie A. The health impacts of a 4-month long community-wide COVID-19 lockdown: Findings from a prospective longitudinal study in the state of Victoria, Australia. PLoS One. 2022;17(4):13. doi:10.1371/journal.pone.0266650.
15. Kavanagh A, Dickinson H, Dimov S, Shields M, McAllister A. The COVID-19 vaccine intentions of Australian disability support workers. Aust NZ J Publ Health. 2022;46(3):314-21. doi:10.1111/1753-6405.13226.
16. Kavanagh A, Hatton C, Stancliffe RJ, Aitken Z, King T, Hastings R, Totsika V, Llewellyn G, Emerson E. Health and healthcare for people with disabilities in the UK during the COVID-19 pandemic. Disabil Health J. 2022;15(1):8. doi:10.1016/j.dhjo.2021.101171.
17. Lima F, O'Donnell M, Bourke J, Wolff B, Gibberd A, Llewellyn G, Leonard H. Child protection involvement of children of mothers with intellectual disability. Child Abuse Negl. 2022;126:10. doi:10.1016/j.chiabu.2022.105515.
18. Olney S, Devine A, Karanikolas P, Dimov S, Malbon J, Katsikis G. Disability and work in a health and economic crisis: Mitigating the risk of long-term labour market exclusion for Australians with disability through policy coordination. Aust J Public Adm. 2022;81(1):163-80. doi:10.1111/1467-8500.12500.
19. Shea B, Bailie J, Dykgraaf SH, Fortune N, Lennox N, Bailie R. Access to general practice for people with intellectual disability in Australia: a systematic scoping review. BMC Prim Care. 2022;23(1):11. doi:10.1186/s12875-022-01917-2.
20. Shields M, Spittal MJ, Dimov S, Kavanagh A, King TL. Employment and disability among young Australians and associations with psychological distress during the COVID-19 pandemic. SSM-Popul Health. 2022;19:4. doi:10.1016/j.ssmph.2022.101140.
21. Wayland S, Newland J, Gill-Atkinson L, Vaughan C, Emerson E, Llewellyn G. I had every right to be there: discriminatory acts towards young people with disabilities on public transport. Disabil Soc. 2022;37(2):296-319. doi:10.1080/09687599.2020.1822784.

### **2023**

1. Aitken Z, Emerson E, Kavanagh AM. COVID-19 vaccination coverage and vaccine hesitancy among Australians with disability and long-term health conditions. Health Promot J Aust. 2023;34(4):895-902. doi:10.1002/hpja.691.
2. Badji S, Kavanagh A, Petrie D. The impact of Disability Insurance reassessment on healthcare use. Health Econ. 2023;32(7):1581-602. doi:10.1002/hec.4680.
3. Bailie J, Fortune N, Plunkett K, Gordon J, Llewellyn G. A call to action for more disability-inclusive health policy and systems research. BMJ Glob Health. 2023;8(3):7. doi:10.1136/bmjgh-2022-011561.
4. Bishop GM, Kavanagh AM, Disney G, Aitken Z. Trends in mental health inequalities for people with disability, Australia 2003 to 2020. Aust N Z J Psych. 2023;57(12):1570-9. doi:10.1177/00048674231193881.
5. Dickinson H, Kavanagh A, Dimov S, Shields M, McAllister A. Political legitimacy and vaccine hesitancy: Disability support workers in Australia. Policy Soc. 2023;42(1):104-16. doi:10.1093/polsoc/puac030.
6. Dickinson H, Smith C, Yates S, Tani M. The importance of social supports in education: survey findings from students with disability and their families during COVID-19. Disabil Soc. 2023;38(8):1304-26. doi:10.1080/09687599.2021.1994371.
7. Dickinson H, Yates S. A decade on: The achievements and challenges of the National Disability Insurance Scheme's implementation. Aust J Soc Iss. 2023;58(3):460-75. doi:10.1002/ajs4.277.
8. Disney G, Petrie D, Yang Y, Aitken Z, Gurrin L, Kavanagh A. Smoking Inequality Trends by Disability and Income in Australia, 2001 to 2020. Epidemiology. 2023;34(2):302-9. doi:10.1097/ede.0000000000001582.
9. Fortune N, Bailie J, Gordon J, Plunkett K, Hargrave J, Madden R, Llewellyn G. Developing self-report disability questions for a voluntary patient registration form for general practice in Australia. Aust NZ J Publ Health. 2023;47(2):8. doi:10.1016/j.anzjph.2023.100032.
10. Kavanagh A, Dickinson H, Dimov S, Shields M, McAllister A. Predictors of vaccine hesitancy among disability support workers in Australia: A cross-sectional survey. Disabil Health J. 2023;16(1):8. doi:10.1016/j.dhjo.2022.101369.
11. Leung XY, Kavanagh AM, Quang QT, Shields M, Aitken Z. A systematic review of the impact of the COVID-19 pandemic on the mental health of adolescents and young people with disabilities aged 15-29 years. BMC Public Health. 2023;23(1):10. doi:10.1186/s12889-023-16260-z.
12. Ma BH, Badji S, Petrie D, Llewellyn G, Chen G. Social interventions to support people with disability: A systematic review of economic evaluation studies. PLoS One. 2023;18(1):18. doi:10.1371/journal.pone.0278930.
13. McAllister A, Dickinson H, Huska M, Devine A, Dimov S, Kavanagh A. 'That was all over the shop': Exploring the COVID-19 response in disability residential settings. Aust J Public Adm. 2023;83(3):271-87. doi:10.1111/1467-8500.12574.
14. Saxby K, Dickinson H, Petrie D, Kavanagh A, Aitken Z. The impact of employment on mental healthcare use among people with disability: distinguishing between part- and full-time employment. Scand J Work Environ Health. 2023;49(8):598-609. doi:10.5271/sjweh.4123.
15. Shields M, Spittal MJ, Aitken Z, Dimov S, Kavanagh A, King TL. Does employment status mediate the association between disability status and mental health among young adults? Evidence from the Household, Income and Labour Dynamics in Australia (HILDA) survey. Occup Environ Med. 2023;80(9):498-505. doi:10.1136/oemed-2023-108853.
16. Smith C, Tani M, Yates S, Dickinson H. Successful School Interventions for Students with Disability During Covid-19: Empirical Evidence from Australia. Asia-Pac Educ Res. 2023;32(3):367-77. doi:10.1007/s40299-022-00659-0.
17. Ye L, Kavanagh A, Petrie D, Dickinson H, Aitken Z. Part-time versus full-time employment and mental health for people with and without disability. SSM-Popul Health. 2023;23:9. doi:10.1016/j.ssmph.2023.101446.

## **2.2 Books**

### **2018**

1. Craven L, Dickinson H, Carey G. Boundary Crossing in Policy and Public Management: Tackling the Critical Challenges. New York: Routledge; 2018.

### **2020**

1. Stancliffe RJ, Wehmeyer ML, Shogren KA, Abery BH. Choice, preference, and disability: Promoting self-determination across the lifespan. Cham, Switzerland: Springer; 2020.

### **2022**

1. Felder F, Davy L, Kayess R. Disability law and human rights: Theory and Policy. Cham, Switzerland: Springer Nature; 2022.
2. Stancliffe RJ, Wiese MY, McCallion P, McCarron M. End of life and people with intellectual and developmental disability: contemporary issues, challenges, experiences and practice. Cham, Switzerland: Springer; 2022.

## **2.3 Book chapters**

### **2016**

1. Hahn JE, Gray J, McCallion P, Ronneberg C, Stancliffe R, Heller T, Janicki M. Transitions in aging: Health, retirement and later life: Review of research, practice and policy. In: Agosta J, Agran M, Ailey S, editors. Critical issues in intellectual and developmental disabilities: Contemporary research, practice, and policy. Washington DC: American Association on Intellectual and Developmental Disabilities; 2016. p. 149-74.
2. Stancliffe R, Arnold S, Riches V. The supports paradigm. In: Schalock R, Keith K, editors. Cross-cultural quality of life: Enhancing the lives of persons with intellectual disability. 2nd ed. Washington DC: American Association on Intellectual and Developmental Disabilities; 2016. p. 133-42.

### **2017**

1. Stancliffe RJ, Brotherton M, O’Loughlin K, Wilson N. Retirement. In: Shogren KA, Wehmeyer ML, Singh NN, editors. Handbook of positive psychology in intellectual and developmental disabilities: Translating research into practice. Washington DC: American Association on Intellectual and Developmental Disabilities; 2017. p. 339-55.

### **2019**

1. Llewellyn G. Parents with disabilities. In: Bornstein M, editor. Handbook of Parenting 3rd ed. New York: Routledge; 2019. p. 234-73.

### **2020**

1. Aunos M, Hodes MW, Llewellyn G, Spencer M, Pacheco L, Janeslätt G, Tarleton B, Springer L, Höglund B. The Choice of Becoming a Parent. In: Stancliffe RJ, Wehmeyer ML, Shogren KA, Abery BH, editors. Choice, Preference, and Disability: Promoting Self-Determination Across the Lifespan. Cham, Switzerland: Springer; 2020. p. 257-81.
2. Curryer B, Dew A, Stancliffe RJ, Wiese MY. Adults with Intellectual Disability: Choice and Control in the Context of Family. In: Stancliffe RJ, Wehmeyer ML, Shogren KA, Abery BH, editors. Choice, Preference, and Disability: Promoting Self-Determination Across the Lifespan. Cham, Switzerland: Springer; 2020. p. 283-302.
3. Llewellyn G. Sexuality and the disregard of lived reality: The sexual abuse of children and young people with disabilities. In: Shuttleworth R, Mona L, editors. The Routledge Handbook of Disability and Sexuality. London: Routledge; 2020. p. 144-57.
4. Stancliffe RJ. Choice availability and people with intellectual disability. In: Stancliffe RJ, Wehmeyer ML, Shogren KA, Abery BH, editors. Choice, preference, and disability: Promoting self-determination across the lifespan. Cham, Switzerland: Springer; 2020. p. 3-26.
5. Stancliffe RJ, Shogren KA, Wehmeyer ML, Abery BH. Policies and practices to support preference, choice, and self-determination: An ecological understanding. In: Stancliffe RJ, Wehmeyer ML, Shogren KA, Abery BH, editors. Choice, preference, and disability: Promoting self-determination across the lifespan. Cham, Switzerland: Springer; 2020. p. 339-54.

### **2021**

1. Emerson E, Aitken Z, Badland H, Fortune N, Green C, Rachele JN. Social and environmental determinants of the health of people with disabilities. In: Putnam M, Bigby C, editors. Handbook on Ageing with Disability. New York: Routledge; 2021. p. 44-56.
2. Kavanagh A, Devine A, Shields M. People with disabilities. In: Detels R, Karim QA, Baum F, Li L, Leyland AH, editors. Oxford Textbook of Global Public Health. 7th ed. Oxford, UK: Oxford University Press Forthcoming; 2021.

### **2022**

1. Forrester-Jones R, McCallion P, McCarron M, Stancliffe RJ, Wiese MY. Accessible funerals and people with intellectual disability. In: Stancliffe RJ, Wiese MY, McCallion P, McCarron M, editors. End of life and people with intellectual and developmental disability: Contemporary issues, challenges, experiences and practice. Cham, Switzerland: Springer; 2022. p. 265-96.
2. Davy L, Green C. The Right to Autonomy and the Conditions that Secure It: The Relationship Between the UNCRPD and Market-Based Policy Reform. In: Felder F, Davy L, Kayess R, editors. Disability Law and Human Rights: Theory and Policy. Cham, Switzerland: Springer; 2022. p. 127-49.
3. Dickinson H, Llewellyn G, Kavanagh A. Examining Australia's performance in realising CRPD obligations in health through the lens of COVID-19. In: Felder F, Davy L, Kayess R, editors. Disability Law and Human Rights: Theory and Policy. Cham, Switzerland: Springer; 2022. p. 239-61.
4. Wiese MY, Stancliffe RJ, McCallion P, McCarron M. Positioning the Issues: An Agenda for Future End-of-Life Research, Policy and Practice. In: Stancliffe RJ, Wiese MY, McCallion P, McCarron M, editors. End of Life and People with Intellectual and Developmental Disability: Contemporary Issues, Challenges, Experiences and Practice. Cham, Switzerland: Springer; 2022. p. 435-63.

## **2.4 Reports**

### **2017**

1. Anderson J, McVilly K, Koritsas S, Johnson H, Wiese M, Stancliffe R, Lyon K, Rezzani N, Ozge J. Accessible written information resources for adults with intellectual disability: Good practice summary. Research to Action Guide. Melbourne: NDS Centre for Applied Disability Research, Swinburne; 2017. Available from: <https://doi.org/10.25916/sut.27923244.v1>
2. Emerson E,  Newland J, Vaughan C, Llewellyn G. Physical violence and property crime reported by people with and without disability NSW 2002-15. Sydney: Centre for Disability Research and Policy, University of Sydney; 2017. Available from: <https://doi.org/10.13140/RG.2.2.17304.08968>
3. Llewellyn G, Emerson E, Smith-Merry J, Gilroy J, Madden R, Kavanagh A. Audit of Disability Research in Australia. Sydney: Centre for Disability Research and Policy, University of Sydney; 2017. Available from: <https://doi.org/10.26188/15153561>
4. Warr D, Dickinson H, Olney S, Hargrave J, Karanikolas A, Kasidis V, Katsikis G, Ozge J, Peters D, Wheeler J, Wilcox M. Choice, Control and the NDIS. Melbourne: University of Melbourne; 2017. Available from: <https://socialequity.unimelb.edu.au/__data/assets/pdf_file/0008/2598497/Choice-Control-and-the-NDIS.pdf>

### **2018**

1. Green C, Carey G, Dickinson H. Debates in Disability and Health Policy: Health inequities and the SDH for people with disability in Australia. Melbourne: Centre of Research Excellence in Disability and Health; 2018. Available from: <https://doi.org/10.26188/16564002>
2. Green C, Malbon E, Carey G, Dickinson H, Reeders D. Competition and collaboration between service providers in the NDIS. Sydney: Centre for Social Impact, UNSW; 2018. Available from: <https://assets.csi.edu.au/assets/research/Competition-and-Collaboration-Between-Service-Providers-in-the-NDIS.pdf>
3. Zhou Q, Llewellyn G, Emerson E, Stancliffe R, Badland H. Spatial distribution of working age adults with disabilities across Australia: a small area analysis of the 2016 Census: Technical Report. Melbourne: Centre of Research Excellence in Disability and Health; 2018. Available from: <https://doi.org/10.26188/13064228>

### **2019**

1. Disney G, Llewellyn G, Shields M, Kavanagh A. The Australian Disability and Health Data Compendium. Melbourne: Centre of Research Excellence in Disability and Health; 2019. Available from: <https://doi.org/10.26188/5c622738d4faf>
2. Kavanagh A, Bollier AM, Katsikis G, Katsidis V, Ozge J. Changing Attitudes: Survey of community attitudes around disability. Melbourne: University of Melbourne and Department of Health and Human Services; 2019. Available from: <https://apo.org.au/node/313845>
3. Rachelle JN, Wiesel I, van Holstein E, de Vries T, Green C, Bicknell E. Making the City of Melbourne more inclusive for people with disability. Melbourne: Melbourne Disability Institute, University of Melbourne; 2019. Available from: <https://figshare.unimelb.edu.au/articles/book/Making_the_City_of_Melbourne_more_inclusive_for_people_with_disability/8189063>
4. Reeders D, Carey G, Malbon E, Dickinson H, Gilchrist D, Duff G, Chand S, Kavanagh A, Alexander D. Market Capacity Framework. Sydney: Centre for Social Impact, UNSW; 2019. Available from: <https://assets.csi.edu.au/assets/research/Market-Capacity-Framework-An-Approach-for-Identifying-Thin-Markets-in-the-NDIS-Report.pdf>

### **2020**

1. Byars S, Sutherland G, Vashishtha R, Bollier AM, Krnjacki L, Hargrave J, Llewellyn G, Kavanagh A. The Australian Disability and Violence Data Compendium. Melbourne: Centre of Research Excellence in Disability and Health; 2020. Available from: <https://doi.org/10.26188/5eb929ebd5aa3>
2. Carey G, Malbon E. Information sharing as market stewardship in the NDIS. Sydney: Centre for Social Impact, UNSW; 2020. Available from: <https://assets.csi.edu.au/assets/research/Information-Sharing-as-Market-Stewardship-in-the-NDIS-Report.pdf>
3. Carey G, Weier M, Malbon E, Duff G, Dickinson H. How is the disability sector faring? A report from the National Disability Services Annual Market Survey. Sydney: Centre for Social Impact, UNSW; 2020. Available from: <https://assets.csi.edu.au/assets/research/How-is-the-Disability-Sector-Faring-Report-2020.pdf>
4. Devine A, Olney S, Mallet S, Dimov S, Katsikis G, Karanikolas A. Exploring the interface between the NDIS and Disability Employment Services (DES). Melbourne: Melbourne Disability Institute, The University of Melbourne; 2020. Available from: <https://disability.unimelb.edu.au/__data/assets/pdf_file/0004/3490267/NDIS-DES-Research-Report-3-September-2020.pdf>
5. Dickinson H, Smith C, Yates S & Bertuo M. Not even remotely fair: Experiences of students with disability during COVID-19 - Full Report. Melbourne: Children and Young People with Disability Australia; 2020. Available from: <http://hdl.handle.net/11343/268204>
6. Emerson E,  Llewellyn G, Stancliffe R, Badland H, Kavanagh A, Disney G,  Zhou Q. A Fair Go? Measuring Australia’s progress in reducing disadvantage for people with disabilities (2001-2016). Melbourne: Centre of Research Excellence in Disability and Health; 2017. Available from: <https://doi.org/10.26188/13064219>
7. Fortune N, Badland H, Clifton S, Emerson E, Rachele J, Stancliffe R, Zhou Q, Lewellyn G. The Disability and Wellbeing Monitoring Framework and Indicators: Technical Report 2020. Melbourne: Centre of Research Excellence in Disability and Health; 2020. Available from: <https://doi.org/10.26188/5e8d144531585>
8. Kavanagh A, Dimov S, Shields M, McAllister A, Dickinson H. Disability support workers: the forgotten workforce in COVID-19. Research Report. Melbourne: Centre of Research Excellence in Disability and Health; 2020. Available from: <https://doi.org/10.26188/12756428>
9. Stancliffe RJ, Badji S, Disney G. Australian Disability Employment Service outcomes in 2020: Effects of COVID-19 restrictions. Melbourne: Centre of Research Excellence in Disability and Health; 2020. Available from: <https://melbourne.figshare.com/articles/online_resource/_/13206494>

### **2021**

1. Aitken Z, Fortune N, Krnjacki L, Badji S, Disney G, Kavanagh A. Methodology Paper: Identification of people with disability in linked administrative data. Melbourne: University of Melbourne; 2021. Available from: <https://www.ndda.gov.au/sites/default/files/documents/2024-09/identification-people-disability-linked-administrative-data-methodology-paper-1.pdf>
2. Devine A, Disney G, Rangi M, Yang Y, Summers P, Huska M, Barney J, Dickinson H, McAllister A, Deane K, Bonyhady B, Kavanagh A. 2021. NDIS Utilisation Project - Victoria, Summary report. Melbourne: Melbourne Disability Institute, University of Melbourne; 2021. ISBN: 978 0 7340 5681 8. Available from: <https://disability.unimelb.edu.au/__data/assets/pdf_file/0019/4081006/MDI-NDIS-Summary-Report-FINAL.pdf>
3. Devine A, Rangi M, Huska M, Barney J, Dickinson H, McAllister A, Bonyhady B, Kavanagh A. NDIS Utilisation Project - Victoria, Qualitative findings report. Melbourne: Melbourne Disability Institute, University of Melbourne; 2021. ISBN: 978 0 7340 5679 5. Available from: <https://disability.unimelb.edu.au/__data/assets/pdf_file/0005/4817138/Vic-NDIS-Utilisation-Project-Qualitative-report-FINAL.pdf>
4. Dickinson H, Yates S, Smith C, Doyle A. Avoiding simple solutions to complex problems: Independent Assessments are not the way to a fairer NDIS. Report prepared for Children and Young People with Disability Australia (CYDA). Melbourne: Public Service Research Group, UNSW; 2021. ISBN: 978-0-6489169-2-5. Available from: <https://cyda.org.au/avoiding-simple-solutions-to-complex-problems-independent-assessments-are-not-the-way-to-a-fairer-ndis-full-report/>
5. Dimov S, Kavanagh A, Shields M, Badji S, LaMontagne A, Vaughan C, Petrie D, King T, Dickinson H. Youth Employment Study: Findings from the first survey. Melbourne: University of Melbourne; 2021. Available from: <https://doi.org/10.26188/14747361>
6. Dimov S, Milner A, Byars S, Devine A, Kavanagh A. Improving Disability Employment Study (IDES): Wave 1 Findings. Melbourne: University of Melbourne; 2021. Available from: <https://doi.org/10.26188/14882544>
7. Disney G, Yang Y, Summers P, Devine A, Deane K, Bonyhady B, Kavanagh A. NDIS Utilisation Project, Victoria. Inequities and causal modelling of plan size and spending. Melbourne: Melbourne Disability Institute, University of Melbourne; 2021. ISBN: 978 0 7340 5681 8. Available from: <https://apo.org.au/node/317263>
8. Fortune N, Bailie J, Gordon J, Madden R, Llewellyn G. Capturing patient disability information in a Voluntary Patient Registration scheme. Rapid review of approaches for capturing disability information. Sydney: Centre for Disability Research and Policy, University of Sydney; 2021.
9. Fortune N, Bailie J, Gordon J, Noti I, Madden R, Llewellyn G. Capturing patient disability information in a Voluntary Patient Registration scheme: consultation report. Sydney: Centre for Disability Research and Policy, University of Sydney; 2021.
10. Fortune N, Bailie J, Gordon J, Noti I, Madden R, Llewellyn G. Getting information about a patient's disability from the Voluntary Patient Registration (VPR) scheme: A short report for everyone who was part of the project. Centre for Disability Research and Policy, The University of Sydney. December 2021
11. Fortune N, Bailie J, Gordon J, Plunkett K, Hargrave J, Madden R, Llewellyn G. Capturing patient disability information in a Voluntary Patient Registration scheme. Final project report. Sydney: Centre for Disability Research and Policy, University of Sydney; 2021.
12. Green C, Carey G, Dickinson H. Barriers and enablers in the development of a COVID-19 policy response for people with disability in Australia. Melbourne: Centre of Research Excellence in Disability and Health; 2021. Available from: <https://doi.org/10.26188/14825457>
13. Griffiths D, Sheehan L, Van Vreden C, Petrie D, Sim M, Collie A. The Community Lockdown in Victoria, Australia (July-October 2020): Implications For Health During The Second COVID-19 Wave. Melbourne: Monash University; 2021. Available from: <https://doi.org/10.26180/13289570>
14. Huska M, Dimov S, Devine A, Dickinson H, Kavanagh A. Managing COVID-19 outbreaks in disability residential settings: Lessons from Victoria's second wave COVID-19. Melbourne: Centre of Research Excellence in Disability and Health; 2021. Available from: <https://doi.org/10.26188/14669568>
15. Bollier AM, Sutherland G, Krnjacki L, Kasidis V, Katsikis G,Ozge J, Kavanagh A. Attitudes Matter: Findings from a National Survey of community attitudes around disability. Melbourne: Centre of Research Excellence in Disability and Health; 2021. Available from: <https://doi.org/10.26188/15176013>
16. Kavanagh A, Dimov S, Shields M, McAllister A, Dickinson H. Disability support workers: COVID-19 vaccine hesitancy and communication needs. Research Report. Melbourne: Centre of Research Excellence in Disability and Health; 2021. Available from: <https://doi.org/10.26188/14456577>
17. Kavanagh A, Dimov S, Shields M, McAllister A, Dickinson H. Disability support workers: the forgotten workforce in COVID-19, Follow up Findings Research Report. Melbourne: Centre of Research Excellence in Disability and Health; 2021. Available from: <https://doi.org/10.26188/14331344.v7>
18. Schilling C, Butterworth P, Petrie D, Saxby K, Harris A, De New S. Mental Health Modelling – Technical Report. Canberra: Commonwealth Department of Health; 2021. Available from: <https://www.pc.gov.au/inquiries/completed/mental-health/report>
19. Schilling C, Butterworth P, Petrie D, Saxby K, Harris A, De New S. Mental Health Modelling – Final Report. Canberra: Commonwealth Department of Health; 2021. Available from: <https://www.pc.gov.au/inquiries/completed/mental-health/report>
20. Sutherland G, Krnjacki L, Hargrave J, Kavanagh A, Llewellyn G, Vaughan C. Primary prevention of violence against women with disability: evidence synthesis. Commissioned research for Respect Victoria. Melbourne: University of Melbourne; 2021. Available from: <https://www.respectvictoria.vic.gov.au/sites/default/files/documents/202105/Disability%20Evidence%20Review.pdf>
21. Weld-Blundell I, Shields M, Davy L, Dickinson H, Kavanagh A, Marck CH. Interventions for employment participation in people with autism, intellectual disability and/or psychosocial disability: a systematic literature review. National Disability Insurance Agency Commissioned Research. Melbourne: University of Melbourne; 2021. Available from: <http://hdl.voced.edu.au/10707/610649>

### **2022**

1. Devine A, Dimov S, LaMontagne A, Vaughan C, Bentley R, Dickinson H, Shields M, Kavanagh A. Improving Disability Employment Study (IDES): End of Study Report. Melbourne: University of Melbourne; 2022. Available from: <https://doi.org/10.26188/19953716.v1>
2. Dimov S, Devine A, Shields M, LaMontagne, A. D., Vaughan C, Bentley R, Dickinson H, Kavanagh A. Improving Disability Employment Study (IDES): Methods of data collection and characteristics of study sample. Melbourne: University of Melbourne; 2022. Available from: <https://doi.org/10.26188/19947206.v1>
3. Doyle C, Dodd S, Dickinson H, Yates S, Buick F. 'There's not just a gap, there's a chasm': The boundaries between Australian disability services and prisons. Canberra: Public Service Research Group, UNSW; 2022. Available from: <https://www.unsw.adfa.edu.au/sites/default/files/documents/The%20boundaries%20between%20Australian%20disability%20services%20and%20prisons%20report_1.pdf>
4. Fortune N, Peters R, Badland H, Emerson E, Stancliffe R, Llewellyn G. The disability and wellbeing monitoring framework: Baseline Indicator Report. Melbourne: Centre of Research Excellence in Disability and Health: Melbourne; 2022. Available from: <https://doi.org/10.25910/ffxs-wd42>

### **2023**

1. Aitken Z, Bishop G, Kavanagh A. Final Report: COVID-19 Vaccine Strategy Project (PO45001493396). Melbourne: Centre of Research Excellence in Disability and Health; 2023.
2. Aitken Z, Kelk AB, Saxby K, Ye L, Petrie D, Kavanagh A, Dickinson H. WISE Employment: The impact of part time work for people with disability. Melbourne: Centre of Research Excellence in Disability and Health; 2022. Available from: <https://www.unsw.adfa.edu.au/sites/default/files/documents/RFQ07019_MSPGH_CRE_DH_Wise_Employment_Report.pdf>
3. Badland H, Kavanagh A, Dickinson H, Dimov S, Bailie J, Sully A, Yates S, Llewellyn G. Melbourne: Centre of Research Excellence in Disability and Health: Impact Evaluation Report, 2016 – 2022. Melbourne: University of Melbourne; 2023. Available from: <https://doi.org/10.26188/21160444>

## **2.5 Factsheets**

### **2018**

1. Aitken Z, Simpson JA, Gurrin L, Bentley R, Kavanagh AM. Does someone’s socioeconomic situation affect their mental health when they acquire a disability? Melbourne: Centre of Research Excellence in Disability and Health; 2018. Available from: <https://credh.org.au/wp-content/uploads/2022/10/27103_CRE-DH-Socioeconomics_Flyer-FA1.pdf>
2. Krnjacki L, Priest N, Aitken Z, Emerson E, Llewellyn G, King T, Kavanagh A. Disability-based discrimination in Australia. Melbourne: Centre of Research Excellence in Disability and Health; 2018. Available from: <https://credh.org.au/wp-content/uploads/2022/10/27093_CRE-DH-Discrimination_Flyer-FA.pdf>
3. Milner A, King TL, LaMontagne AD, Aitken Z, Petrie D, & Kavanagh A. Underemployment and mental health of people with disability. Melbourne: Centre of Research Excellence in Disability and Health; 2018. Available from: <https://credh.org.au/wp-content/uploads/2022/10/27162-CRE-DH_Underemployment_Summary_A4.pdf>

### **2019**

1. Aitken Z. Precariously Placed: Housing circumstances for Australians with disability. Melbourne: Centre of Research Excellence in Disability and Health; 2019. Available from: <https://doi.org/10.26188/5ce4bfc332320>
2. Kavanagh AM, Priest N, Emerson E, Milner A, King T. Bullying by adolescents with and without a disability. Melbourne: Centre of Research Excellence in Disability and Health; 2019. Available from: <https://credh.org.au/publications/old/the-experience-of-bullying-from-an-adolescent-perspective/>
3. Aitken Z, Simpson JA, Gurrin L, Bentley R, Kavanagh AM. Does someone’s socioeconomic situation affect their mental health when they acquire a disability? Melbourne: Centre of Research Excellence in Disability and Health; 2019. Available from: <https://credh.org.au/wp-content/uploads/2022/10/27103_CRE-DH-Socioeconomics_Flyer-FA1.pdf>
4. 1Zhou Q, Llewellyn G, Emerson E, Badland H, Stancliffe R. Where do people with disabilities live across Australia. Melbourne: Centre of Research Excellence in Disability and Health; 2019. Available from: <https://doi.org/10.26188/5c75f384c4502>

### **2020**

1. Emerson E, Llewellyn G, Badland H, Stancliffe R, Kavanagh A, Disney G, Zhou, Q. A Fair Go? What progress have we made in reducing inequality for people with disabilities. Melbourne: Centre of Research Excellence in Disability and Health; 2020. Available from: <https://doi.org/10.26188/13064219.v1>
2. Sutherland G, Bollier AM, Krnjacki L, Llewellyn G, Kavanagh A. Violence against people with disability in Australia. Melbourne: Centre of Research Excellence in Disability and Health; 2020. Available from: <https://doi.org/10.26188/12935762.v2>
3. Sutherland G, Bollier AM, Krnjacki L, Llewellyn G, Kavanagh A. Violence against young people with disability in Australia. Melbourne: Centre of Research Excellence in Disability and Health; 2020. Available from: <https://doi.org/10.26188/12756425.v2>
4. Sutherland G, Bollier AM, Krnjacki L, Llewellyn G, Kavanagh A. Violence against people with disability in Australia – by impairment. Melbourne: Centre of Research Excellence in Disability and Health; 2020. Available from: <https://doi.org/10.26188/12991526.v2>
5. Sutherland G, Bollier AM, Krnjacki L, Llewellyn G, Kavanagh A. Intimate partner violence against people with disability in Australia. Melbourne: Centre of Research Excellence in Disability and Health; 2020. Available from: <https://doi.org/10.26188/12991586.v3>
6. McAllister A, Dimov S, Shields M, Dickinson H, Kavanagh A. Disability Support Workers: The forgotten workforce during COVID-19, Keeping DSWs and People with Disability Safe, Fact Sheet 1. Melbourne: Centre of Research Excellence in Disability and Health; 2020. Available from: <https://doi.org/10.2618/12756425.v2>
7. McAllister A, Dimov S, Shields M, Dickinson H, Kavanagh A. Disability Support Workers: The forgotten workforce during COVID-19, Financial and Psychological Impacts of COVID-19, Fact Sheet 2. Melbourne: Centre of Research Excellence in Disability and Health; 2020. Available from: <https://doi.org/10.26188/12756425.v2>

### **2021**

1. Fortune N, Stancliffe R, Emerson E, Llewellyn G, Kavanagh A. Disadvantage facing young people with disability in Australia: what has changed over time? Melbourne: Centre of Research Excellence in Disability and Health; 2021. Available from: <https://credh.org.au/projects/social-determinants/disadvantage-and-young-people-with-disability/>
2. Green C, Dickinson H, Carey G. Debates in Disability and Health Policy: What are the policy gaps for people with disability? Melbourne: Centre of Research Excellence in Disability and Health; 2021. Available from: <https://figshare.unimelb.edu.au/articles/online_resource/Debates_in_Disability_and_Health_Policy/16564002/2>
3. Dickinson H. Policy Lab, Delphi Study Explainer. Melbourne: Centre of Research Excellence in Disability and Health; 2021. Available from: <https://credh.org.au/wp-content/uploads/2024/07/CRE-DH-Policy-Lab-Explainer-1.pdf>

**Appendix 3: CRE-DH Workshops, Forums and Seminars**

| **Date** | **Capacity Strengthening Activity** | **Delivery Mode & Attendance Data (where available)** | **Assumptions** |
| --- | --- | --- | --- |
| 27 June 2017 | National launch of CRE-DH & panel discussion - What makes us healthy? A disability perspective | Face-to-face  1700 views of video highlights on YouTube | Not costed as no attendance numbers available |
| 28 May 2018 | Melbourne Disability Institute (MDI) launch | Hybrid and recorded  183 views of recording on YouTube | Not costed - mostly online |
| 19 July 2018 | Seminar - Empowering people with disabilities without exploiting support workers – CRE-DH & MDI | Hybrid | Not costed - mostly online |
| 13 May 2019 | Panel discussion - Precariously placed: Housing, health and disability in Australia | Face-to-face  80 attendees | Costed. 80 attendees x 2 hours x $Senior Research Fellow wage + oncosts |
| 18 July 2019 | Policy forum - Connecting policy and research in disability and health | Face-to-face  29 attendees from 16 organisations (Commonwealth government departments, Commonwealth statutory agencies, and Disability Representative Organisations) | Costed. 29 attendees x 2 hours x $Senior Research Fellow wage + oncosts |
| 26 May 2020 | Webinar - Information accessibility for people with disability during the COVID-19 crisis - CRE-DH & UNSW Disability Innovation Institute | Online  101 views of recording on YouTube | Not costed - online. |
| 23 September 2020 | Panel discussion - Disruption and Disability: Sydney Ideas | Face-to-face  1747 plays of recording SoundCloud | Not costed as no attendance numbers available |
| 24 September 2020 | Panel discussion - National Disability Strategy: Beyond 2020 - CRE-DH & Australian Human Rights Commission | Online  1300 views of recording on YouTube | Not costed - online |
| 24 August 2022 | Webinar - How can we use quantitative data for disability research? | Online  301 attendees  133 views of recording on YouTube | Not costed - online |
| 15 & 16 September 2022 | Policy forum - CRE-DH Policy Forum (2 days) | Online  Attended by 32 representatives from 15 organisations (Commonwealth government departments, Commonwealth statutory agencies, and Disability Representative Organisations) | Costed as we have attendee data. 32 attendees x 2 hours x 2 days x $Senior Research Fellow wage + oncosts |
| 29 March 2023 | Webinar - The value of part time work for Disability Employment Service participants - CRE-DH & WISE Employment | Online  201 views of recording on YouTube | Not costed – online |

Note: Views on YouTube as at 16/04/2025
